# Supplementary material for: Olive fruit fly and its obligate symbiont Candidatus Erwinia dacicola: Two new symbiont haplotypes in the Mediterranean basin
Source: PLoS One. 2021 Sep 8;16(9):e0256284. doi: 10.1371/journal.pone.0256284 (PMC8425570; doi:10.1371/journal.pone.0256284)
Supplement: S1 Fig — (DOCX) [file pone.0256284.s001.docx]

**S4 Fig. Bactrocera oleae maximum likelihood reconstruction**


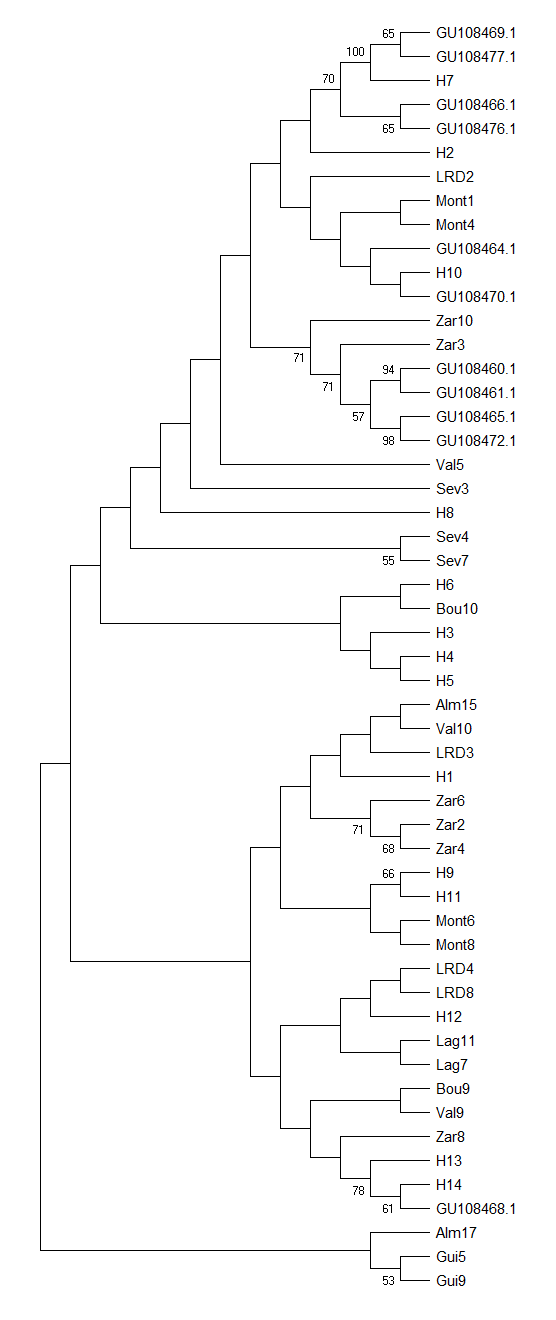


**Figure**: Evolutionary history inferred by using the Maximum Likelihood method and Kimura 2-parameter model [1]. Only bootstrap value higher than 70% (in 1000 replicates) are shown. Evolutionary analyses were conducted in MEGA X [2]. Details on the main text.

1. Kimura M. (**1980**). A simple method for estimating evolutionary rate of base substitutions through comparative studies of nucleotide sequences. *Journal of Molecular Evolution* **16**:111-120.

2. Kumar S., Stecher G., Li M., Knyaz C., and Tamura K. (**2018**). MEGA X: Molecular Evolutionary Genetics Analysis across computing platforms. *Molecular Biology and Evolution* **35**:1547-1549.

3. Felsenstein J. (**1985**). Confidence limits on phylogenies: An approach using the bootstrap. *Evolution* **39**:783-791.
