# Supplementary material for: Olive fruit fly and its obligate symbiont Candidatus Erwinia dacicola: Two new symbiont haplotypes in the Mediterranean basin
Source: PLoS One. 2021 Sep 8;16(9):e0256284. doi: 10.1371/journal.pone.0256284 (PMC8425570; doi:10.1371/journal.pone.0256284)
Supplement: Attachment — Submitted filename: PONE-D-21-11736_R1.pdf [file pone.0256284.s008.pdf]

PLOS ONE

Olive fruit fly and its obligate symbiont Candidatus Erwinia dacicola: two new symbiont haplotypes in the Mediterranean basin

--Manuscript Draft--

**Manuscript Number:**	PONE-D-21-11736R1
**Article Type:**	Research Article
**Full Title:**	Olive fruit fly and its obligate symbiont Candidatus Erwinia dacicola: two new symbiont haplotypes in the Mediterranean basin
**Short Title:**	Ca. E. dacicola, two new haplotypes in the Mediterranean basin
**Corresponding Author:**	Tânia Nobre<br>Universidade de Evora<br>Évora, PORTUGAL
**Keywords:**	pest management; olive pest; Bactrocera oleae; holobiont; symbiont transmission; Ca. Erwinia dacicola
**Abstract:**	The olive fruit fly, specialized to become monophagous during several life stages, remains the most important olive tree pest with high direct production losses, but also affecting the quality, composition, and inherent properties of the olives. Thought to have originated in Africa is nowadays present wherever olive groves are grown. The olive fruit fly evolved to harbor a vertically transmitted and obligate bacterial symbiont - Candidatus Erwinia dacicola- leading thus to a tight evolutionary history between olive tree, fruit fly and obligate, vertical transmitted symbiotic bacterium. Considering this linkage, the genetic diversity (at a 16S fragment) of this obligate symbiont was added in the understanding of the distribution pattern of the holobiont at nine locations throughout four countries in the Mediterranean Basin. This was complemented with mitochondrial (four mtDNA fragments) and nuclear (ten microsatellites) data of the host. We focused on the previously established Iberian cluster for the B. oleae structure and hypothesised that the Tunisian samples would fall into a differentiated cluster. From the host point of view, we were unable to confirm this hypothesis. Looking at the symbiont, however, two new 16S haplotypes were found exclusively in the populations from Tunisia. This finding is discussed in the frame of host-symbiont specificity and transmission mode. To understand olive fruit fly population diversity and dispersion, the dynamics of the symbiont also needs to be taken into consideration, as it enables the fly to, so efficiently and uniquely, exploit the olive fruit resource.
**Order of Authors:**	Tânia Nobre
**Opposed Reviewers:**	
**Response to Reviewers:**	Reviewer #1: The author followed a holistic approach combining data from both the host and the endosymbiont. Interestingly two new 16S haplotypes regarding the Candidatus Erwnia dacicola were detected for the Tunisian olive fly populations, which is the major finding of the manuscript, adding valuable data in the existing findings regarding the endosymbiont genetic variability. There is also an interesting overall commentary regarding the olive fly population diversity and coevolution with the symbiont.<br><br>Generally, the manuscript is well written and structured employing a wide list of genomic tools and approaches. There is a main disadvantage regarding the microsatellites part with a restricted number of flies used for the analysis in order to have a reliable estimation. Author has explained the reason behind this and therefore assumptions based on microsatellites throughout the text are minimum.<br><br>There is a short list of minor issues that I would like to be addressed through a revised version of the manuscript:<br><br>A supplemental list with the primers/probes used would be beneficial for the reader. This list was added as supplemental file.<br><br>Please do not use “e.g.” for citing references within the text.

Powered by Editorial Manager® and ProduXion Manager® from Aries Systems Corporation

Removed.

Line 87-88: “These changes will lead to species specific dynamics with impact on the interaction of the olive and its obligate olive fruit fly”. This is a strong statement and it should be followed by a list of relevant references.
Three relevant references were added to the text, that show that climate change will impact differently different insect species.

Line 249: “following the parsimony” please explain the meaning here and if necessary add references to support it.
The sentence was changed for better accuracy. What was meant as the principle of parsimony was the principle that the simplest explanation is usually the best one.

Line 270: correct “this bacteria was found”
Corrected

Line 287: “uniquely exploit this resource” where do you refer by “resource”?
By resource I meant the fruit of the olive tree. Sentence was changed accordingly

Line 287: please correct “all data suggests” to “all data suggest”.
Corrected

Reviewer #2: PONE-D-21-11736: Olive fruit fly and its obligate symbiont Candidatus Erwinia dacicola: two new symbiont haplotypes in the Mediterranean basin

This paper identifies two new haplotypes of the olive fly symbiont, Ca. Erwinia dacicola, found to be uniquely associated with flies from Tunisia, and significantly different from previously described haplotypes associated with samples from Europe. These findings contrast analyses based on genomic and mitochondrial markers suggesting that the populations sampled from Africa and Europe cluster with no clear association to their geographic location. The main conclusion is that since the symbiont is vertically transmitted and essential to its host the new bacterial linages may contribute to understanding the dispersion patterns and population diversity of the fly.

The paper is well written but please note that there are many remaining typos, syntax and parsing issues which need to be attended. From the perspective of my field of expertise I have only a few comments, mostly concerning topics addressed in the results and discussion. Please make sure that the sequencing data underlying the findings will be made publically available (I couldn’t find a note on this in the manuscript).

The sequencing data on the olive fruit fly are directly available from the S1 Table as it shows all the nucleotide variability in relation to Bactrocera oleae mitochondrion, complete genome AY210702 as reference. The four Ca. Erwinia dacicola 16S haplotypes are also derived from the data made available on S3 File and were also submitted to GenBank under the codes htA: MW888710, htD: MW888711, htB: MW888712 and htC: MW888713.

Introduction:

Page 4, Lines 83-84 and 222-224 (discussion): please state the hypotheses of this study in the introduction as well.
Thank you. This was done and can now be read on Lines 79-80.

Results:
1. Figure 1 depicts the median-joining network of haplotypes derived from mitochondrial DNA analysis. In addition to the populations sampled in this study (9 locations), other flies seem to have been included in the analysis (e.g. H, GU, orange coded), which do not seem to be mentioned in the text. Please include a description of these samples. Mont = MON?
Thanks for the comment, which highlights that the information might not have been clear. The other sequences included are referred in the methodology L-119 to L-121 and these are represented by the GenBank codes and H# represents a haplotype shared by different samples. This information is indeed only presented in (S1 Table)

Powered by Editorial Manager® and ProduXion Manager® from Aries Systems Corporation

	and the haplotypes needed to be derived. Therefore, I added a S2 Table, facilitating the interpretation. A sentence is added to the legend now, for clarification. Mont = MON (now corrected).<br><br>2. In Figure 2 the color codes for Montpelier and Bouficha seem to have been mixed (they do not match those in figure 1). Should there be a representation of the orange-coded cluster as well (as in figure 1)?<br>You are absolutely correct as to the color codes of MON and BOU. Thank you. They are now corrected. Unfortunately, no representative of orange-coded cluster from figure 1 was available in the sampled specimens (they all corresponded to sequences coming from previous study) and therefore they could not be included in the microsatellite analyses.<br><br>3. The STRUCTURE analysis and figure 3: this is a bit confusing. Should the microsatellite-based dendogram depict two clusters (as currently presented) or only one cluster? Please include a short clarification for readers which are unfamiliar with the technicalities of this specific test.<br><br>The UPGMA dendogram by definition depicts a dichotomous structure whereas STRUCTURE infers the most likely number of clusters for a given data set using a Bayesian approach. A sentence was added to aid the reader, as suggested.<br><br>Discussion:<br>The discussion addresses several issues but is currently presented as one continuous mass of text. I suggest to divide the text into clear sections complying with the addressed subjects.<br>Thank you for the suggestion. I did struggle with finding clear sections complying with the results addressed because my original effort was exactly the opposite: I have attempted to give a more holistic perspective to the discussion by combining data from both the host and the endosymbiont. I do believe that this is one of the strong points of the manuscript, also recognized by reviewer one. Therefore, I decided to stay with my original approach.<br><br>Lines 245 – 248: according to file S3, flies sampled from Tunisia (and other locations as well) can be associated with either one of the 4 identified symbiont haplotypes. Two points which might be considered to be included in the discussion: (1) Can the above be congruent with these two hypotheses?, and (2) can certain fly genotypes (e.g. mitochondrial haplotypes) be more prone to be associated with a certain bacterial haplotype?<br>No doubt are the points raised interesting and relevant. Unfortunately, the scarce geographical data available does not allow to dismiss none of the two hypotheses raised in the main text. Also, the interesting possibility of high host-symbiont haplotype specificity is not possible to address with the current data. However, as soon as more data is available, I believe these suggestions can and should be discussed. A sentence was added to the discussion to allocate this comment and the data limitation.<br><br>Line 268: I would separate the evolution of Erwinia into an obligate symbiont from the domestication of the olive. The transition into an obligate symbiont probably occurred far into the evolutionary history of the fly, and is not associated with recent human activity.<br>This was presented in the discussion as the main hypothesis, in lines 264-267. However, it seems that it was not clear enough and therefore a sentence was added for clarification. Also further on the sentence was re-written to accommodate the comment and becoming clearer.<br><br>Lines 269 – 273: another possible explanation contributing to retention of genome size of the Erwinia symbiont: it is a gut bacterium, and as such it remains exposed to transient bacteria which are ingested with the diet (probably mainly at the adult stage), and thus, to horizontal gene transfer. The composition of the gut microbiome of this fly suggest that bacteria other than the Erwinia symbiont (e.g. free living Enterobacter spp.) are intermittently associated with the fly and may contribute to DNA exchange. This hypothesis is now stated in a clearer way (Line 276-279).
**Additional Information:**	

Powered by Editorial Manager® and ProduXion Manager® from Aries Systems Corporation

Question	Response
**Financial Disclosure**<br><br>Enter a financial disclosure statement that describes the sources of funding for the work included in this submission. Review the submission guidelines for detailed requirements. View published research articles from *PLOS ONE* for specific examples.<br><br>This statement is required for submission and **will appear in the published article** if the submission is accepted. Please make sure it is accurate.<br><br>**Unfunded studies**<br>Enter: *The author(s) received no specific funding for this work.*<br><br>**Funded studies**<br>Enter a statement with the following details:<br>• Initials of the authors who received each award<br>• Grant numbers awarded to each author<br>• The full name of each funder<br>• URL of each funder website<br>• Did the sponsors or funders play any role in the study design, data collection and analysis, decision to publish, or preparation of the manuscript?<br>• **NO** - Include this sentence at the end of your statement: *The funders had no role in study design, data collection and analysis, decision to publish, or preparation of the manuscript.*<br>• **YES** - Specify the role(s) played.<br><br>* typeset	This work is supported by National Funds through FCT - Foundation for Science and Technology under the research project PTDC/ASP-PLA/30650/2017. The funders had no role in study design, data collection and analysis, decision to publish, or preparation of the manuscript.
**Competing Interests**<br><br>Use the instructions below to enter a competing interest statement for this submission. On behalf of all authors, disclose any competing interests that could be perceived to bias this work—acknowledging all financial support and any other relevant financial or non-financial competing interests.	The authors have declared that no competing interests exist.

This statement is required for submission and **will appear in the published article** if the submission is accepted. Please make sure it is accurate and that any funding sources listed in your Funding Information later in the submission form are also declared in your Financial Disclosure statement.<br><br>View published research articles from *PLOS ONE* for specific examples.<br><br>**NO authors have competing interests**<br><br>Enter: *The authors have declared that no competing interests exist.*<br><br>**Authors with competing interests**<br><br>Enter competing interest details beginning with this statement:<br><br>*I have read the journal's policy and the authors of this manuscript have the following competing interests: [insert competing interests here]*<br><br>* typeset	
**Ethics Statement**<br><br>Enter an ethics statement for this submission. This statement is required if the study involved:<br><br>• Human participants<br>• Human specimens or tissue<br>• Vertebrate animals or cephalopods<br>• Vertebrate embryos or tissues<br>• Field research<br><br>Write "N/A" if the submission does not require an ethics statement.<br><br>General guidance is provided below. Consult the submission guidelines for detailed instructions. **Make sure that all information entered here is included in the Methods section of the manuscript.**	N/A

**Format for specific study types**<br><br>**Human Subject Research (involving human participants and/or tissue)**<br>• Give the name of the institutional review board or ethics committee that approved the study<br>• Include the approval number and/or a statement indicating approval of this research<br>• Indicate the form of consent obtained (written/oral) or the reason that consent was not obtained (e.g. the data were analyzed anonymously)<br><br>**Animal Research (involving vertebrate animals, embryos or tissues)**<br>• Provide the name of the Institutional Animal Care and Use Committee (IACUC) or other relevant ethics board that reviewed the study protocol, and indicate whether they approved this research or granted a formal waiver of ethical approval<br>• Include an approval number if one was obtained<br>• If the study involved *non-human primates*, add *additional details* about animal welfare and steps taken to ameliorate suffering<br>• If anesthesia, euthanasia, or any kind of animal sacrifice is part of the study, include briefly which substances and/or methods were applied<br><br>**Field Research**<br><br>Include the following details if this study involves the collection of plant, animal, or other materials from a natural setting:<br>• Field permit number<br>• Name of the institution or relevant body that granted permission	
**Data Availability**<br><br>Authors are required to make all data underlying the findings described fully available, without restriction, and from the time of publication. PLOS allows rare exceptions to address legal and ethical concerns. See the PLOS Data Policy and FAQ for detailed information.	Yes - all data are fully available without restriction

Powered by Editorial Manager® and ProduXion Manager® from Aries Systems Corporation

A Data Availability Statement describing where the data can be found is required at submission. Your answers to this question constitute the Data Availability Statement and **will be published in the article**, if accepted.<br><br>**Important:** Stating 'data available on request from the author' is not sufficient. If your data are only available upon request, select 'No' for the first question and explain your exceptional situation in the text box.<br><br>Do the authors confirm that all data underlying the findings described in their manuscript are fully available without restriction?	
**Describe where the data may be found in full sentences. If you are copying our sample text, replace any instances of XXX with the appropriate details.**<br><br>• If the data are **held or will be held in a public repository**, include URLs, accession numbers or DOIs. If this information will only be available after acceptance, indicate this by ticking the box below. For example: *All XXX files are available from the XXX database (accession number(s) XXX, XXX.).*<br>• If the data are all contained **within the manuscript and/or Supporting Information files**, enter the following: *All relevant data are within the manuscript and its Supporting Information files.*<br>• If neither of these applies but you are able to provide **details of access elsewhere**, with or without limitations, please do so. For example:<br><br>*Data cannot be shared publicly because of [XXX]. Data are available from the XXX Institutional Data Access / Ethics Committee (contact via XXX) for researchers who meet the criteria for access to confidential data.*<br><br>*The data underlying the results presented in the study are available*	All relevant data are within the manuscript and Supporting Information files.

from (include the name of the third party and contact information or URL).<br>• This text is appropriate if the data are owned by a third party and authors do not have permission to share the data.<br><br>* typeset	
Additional data availability information:	Tick here if the URLs/accession numbers/DOIs will be available only after acceptance of the manuscript for publication so that we can ensure their inclusion before publication.

Powered by Editorial Manager® and ProduXion Manager® from Aries Systems Corporation

Dear editor,

Herewith I re-submit the manuscript “Olive fruit fly and its obligate symbiont *Candidatus* Erwinia dacicola: two new symbiont haplotypes in the Mediterranean basin” to be considered for publication in PloS One. Thank you and the reviewers for the comments on the manuscript. I have revised the text, according to the recommendations of the reviewers, discussing the issues raised also in the manuscript. We hope that our revised manuscript can now be published in PloS One.

The present research article deals with the olive fruit fly, generally considered the most important olive tree pest. The fly evolved to harbor a vertically transmitted and obligate bacterial symbiont -*Candidatus* Erwinia dacicola- thought to allow the host to exploit the olive fruit immature stages and completing more than one generation in a crop year. The to date knowledge on this pest population structure did not include data on its obligate symbiont. With the rationality that to understand olive fruit fly population diversity and dispersion, the dynamics of the symbiont should be considered, a first survey took place covering nine locations at four countries in the Mediterranean Basin by analyzing host (at four mtDNA fragments and at 10 microsatellite loci) and symbiont (at a 16S fragment) diversity. The geographical data on the *Ca.* E. dacicola is still scarce and thus the present work duplicates the known number of 16S haplotypes. This diversity is discussed in the frame of host-symbiont specificity and transmission mode.

The relevance and particularities of this intricate tripartite relation between olive tree, olive fruit fly and *Ca*. Erwinia dacicola symbiont, makes the current findings relevant in shaping new research aims and approaches towards understanding the spreading of this pest in a changing environment. Also, it clear connects with the ongoing trend to develop new sustainable pest management methods based on the symbiosis as a highly specific target.

Yours sincerely,

Tânia Nobre

Manuscript

Click here to access/download;Manuscript;Revised Manuscript.docx

Olive fruit fly and its obligate symbiont *Candidatus* Erwinia dacicola: two new symbiont haplotypes in the Mediterranean basin

Tânia Nobre*

MED - Mediterranean Institute for Agriculture, Environment and Development, Instituto de Investigação e Formação Avançada, Universidade de Évora, Pólo da Mitra, Ap. 94, 7006-554 Évora, Portugal.

* Corresponding author

Email: tnobre@uevora.pt

Abstract

The olive fruit fly, specialized to become monophagous during several life stages, remains the most important olive tree pest with high direct production losses, but also affecting the quality, composition, and inherent properties of the olives. Thought to have originated in Africa is nowadays present wherever olive groves are grown. The olive fruit fly evolved to harbor a vertically transmitted and obligate bacterial symbiont -*Candidatus* Erwinia dacicola- leading thus to a tight evolutionary history between olive tree, fruit fly and obligate, vertical transmitted symbiotic bacterium. Considering this linkage, the genetic diversity (at a 16S fragment) of this obligate symbiont was added in the understanding of the distribution pattern of the holobiont at nine locations throughout four countries in the Mediterranean Basin. This was complemented with mitochondrial (four mtDNA fragments) and nuclear (ten microsatellites) data of the host. We focused on the previously established Iberian cluster for the *B. oleae* structure and hypothesised that the Tunisian samples would fall into a differentiated cluster. From the host point of view, we were unable to confirm this hypothesis. Looking at the symbiont, however, two new 16S haplotypes were found exclusively in the populations from Tunisia. This finding is discussed in the frame of host-symbiont specificity and transmission mode. To understand olive fruit fly population diversity and dispersion, the dynamics of the symbiont also needs to be taken into consideration, as it enables the fly to, so efficiently and uniquely, exploit the olive fruit resource.

Keywords: pest management; olive pest; *Bactrocera oleae*; holobiont; symbiont transmission

Introduction

The olive tree was likely the first domesticated fruit tree, and although domestication probably began in the Eastern Mediterranean, selection for cultivars took place at several different independent locations [1–3]. Given time and appropriate conditions, the olive fruit fly evolved to explore this

resource. This fruit fly, *Bactrocera oleae* (Rossi, 1790), has specialized to become monophagous and it remains the most important olive tree pest. Production losses are estimated on an average of more than 15% yearly [4], and this fly has been responsible for losses of up to 80% of oil value and 100% of some table cultivars [5]. To cope with the olive-plant abundant secondary metabolites, particularly the defensive compound oleuropein (a bitter and otherwise toxic phenolic glycoside) [6], the olive fruit fly evolved to harbor a vertically transmitted and obligate bacterial symbiont—*Candidatus* Erwinia dacicola [7] leading thus to a tight evolutionary history between olive tree, fruit fly and obligate, vertical transmitted symbiotic bacterium.

The olive fruit fly is thought to have originated in Africa and then spread to the Mediterranean basin and South Central Asia [8], and it is nowadays present wherever olive groves are grown. Nardi and co-workers [9] presented the first comprehensive study on olive fly populations and found evidence of a population subdivision into three regional groups, Pakistan, Africa and Mediterranean plus America. Further, there is evidence of a separation between Iberian (Western Mediterranean) and Italic (Central Mediterranean) olive fly populations [10], with no clearly defined boundary [8]. Van Asch and co-workers [8] observed intermixing to extend at variable levels throughout their whole studied area, from Northwestern Italy to Portugal.

As an obligate symbiont, mechanisms that ensure vertical transmission have evolved and *Ca.* Erwinia dacicola is transmitted to the following generation at the oviposition [7,11,12] guaranteeing symbiont acquisition by the offspring but also host-symbiont specificity. When transmission is strictly vertical, host switching would not be expected, and co-evolution of host and symbiont lineages should be observed. For both symbiont and host, failure in vertical transmission has high costs, and is usually equivalent to death of the symbiont and significant reduction in host fitness or high mortality. Selection at these endosymbiotic bacteria likely results from selection imposed by the host and that from selection emerging in a symbiotic context but independent from the host [13]. Furthermore, the cyclic events of transmission -where a given number of symbionts are passed to the next generation- are likely to create strong bottlenecks affecting symbiont population dynamics. Not much is known on the diversity and

structure of *ca*. Erwinia dacicola except that there is evidence of two lineages of this endosymbiont in Italian populations, based on 16S rRNA gene sequences [14].

Understanding how the pest population spreads is essential for its management. The close association of this monophagous fly species with its host, and its possibility of laying eggs throughout most of the year, allows for a fairly stable population and the rate of population change is mainly shaped by density-dependent population feedback [15]. Its spreading is intricately connected to the spreading of the plant host, as this relation seems the result of long-term co-evolution. In most areas where olives are grown commercially, the olive fruit fly can be found and have become a major pest. In North America was first detected in 1998 in California [16], and it has spread throughout the region, including in Central and South America. Nowadays it seems that the olive fruit fly can also be found well outside the Mediterranean basin in Central and South Africa, and not only in the Middle East but also in China and India; Australia seems to be the only area in the world, where olives are cultivated and well established for commercial purposes, which is still free of olive fruit fly [17].

Having as rationality this intricate tripartite relation between olive tree, olive fruit fly and *Ca*. Erwinia dacicola symbiont, this work aimed to add this obligate symbiont genetic diversity in the understanding of the distribution pattern of the holobiont at nine locations throughout four countries in the Mediterranean Basin. We focused on the Iberian cluster and on the hypothesis that the Tunisian samples would fall into a differentiated cluster due to the proximity to the Italic peninsula cluster. To date, and to the best of our knowledge, the genetic diversity present within the olive fly symbiont has only investigated for the Italian populations [14]. Therefore, the present study looked at a) haplotypes from four mitochondrial DNA fragments of the host fly, b) patterns of ten microsatellites of the host fly and c) 16S rRNA gene sequence diversity of *Ca.* E. dacicola. Albeit with a limited geographical and populational coverage, it was intended to look for population differentiation, gaining insights into gene flow and spreading. Overall rising temperatures, warmer winter minimum temperatures and changes in precipitation patterns that are likely linked to water shortages [18] are affecting the spreading of insects and their aggressiveness as pests in largely unknown ways [19]. These changes will lead to species

specific dynamics [20–22], which likely will impact on the interaction of the olive and its obligate olive fruit fly.

Methodology

Data collection

Olive fruit flies were sampled at 9 locations (3 in Portugal, 3 in Spain, 1 in France and 2 in Tunisia; Table 1), either directly, with the permission of the owners, or made available by colleagues and/or landowners (see acknowledgements). Sampling did not involve endangered or protected species. Olives were collected and stored in plastic boxes, with emerging pupae and adults being gathered up to 12 specimens per location. Individuals were stored at -20 °C in 70 % ethanol until DNA extraction. Individuals were allowed to dry on filter paper prior to DNA extraction. DNA from whole body tissue was extracted following extraction protocols [23] using CTAB extraction buffer after being ground up with a plastic pestle. Proteins were removed with 24 : 1 isoamylalcohol : chloroform, and DNA precipitated with isopropanol. DNA extracts were eluted in 50 µl of sterile water. All extraction products were stored at -20 °C and later used directly in the PCRs.

Table 1: Sampling locations and collection year (see acknowledgements for collectors' information)

Country	Location	Code	Coordinates		Year
Portugal	Guimarães	GUI	41.46N	8.31W	2016
Portugal	Valverde	VAL	38.86N	7.27W	2019
Portugal	Lagos	LAG	37.13N	8.68W	2016
Spain	LaRoda	LRD	39.09N	2.19W	2019
Spain	Seville	SEV	37.47N	5.99W	2019
Spain	Almeria	ALM	36.89N	2.44W	2016
France	Montpellier	MON	43.61N	3.87E	2017
Tunisia	Bouficha	BOU	36.30N	10.45E	2017
Tunisia	Zarzis	ZAR	33.50N	11.12E	2017

Olive fruit fly mtDNA sequences

Four highly variable sections of mtDNA were amplified and sequenced. These were amongst the five sections selected by van Asch and co-workers [10], based on the number of polymorphisms previously

described in a Mediterranean region dataset [24]. These include both tRNA-Leu genes and segments of the ND2, ND4, COX1 and COX2 and genes, ca. 24% of the complete mitochondrial genome of *B. oleae* [10] (see S1 Table for primer information). PCR reactions were conducted using 1 µl of the extracted DNA in a standard 25 µl reaction, with 0.5 pmol/µl of each primer, 1.5 mM $MgCl^2$, 0.5 mM dNTPs and 0.04 U/ml Taq DNA polymerase. The touch-down cycle protocol involved two-phases; 1) initial denaturation at 94 °C for 5 min, followed by 10 cycles of denaturation at 94 °C for 30 s, annealing at variable temperatures for 30 s (set at 60 °C and decreasing by 0.5 °C per cycle) and extension at 72 °C for 1 min; 2) 25 cycles of 94°C for 30 s, 50 °C for 30 s, and 72°C for 1 min, followed by a final extension of 72 °C for 7 min. The PCR products, after visualized on agarose gel, were purified using the NZYGelpure kit (from NZYTech, Lda) and sequencing was done commercially (Macrogen Inc.).

Electropherograms were inspected using Genestudio V.2.2.0.0 (www.genestudio.com) and sequences were cropped to the minimal region of overlap for all individuals using the same software. The 21 *Bactrocera oleae* full mitochondrial genomes made available by Nardi and co-workers [24] were aligned with our sequences and the same regions of overlap were extracted. All alignments were made using the Muscle tool implemented in MEGA X [25]. The amplified mitochondrial DNA segments and the database obtained ones were concatenated per individual. Median-joining networks [26] were calculated using the PopART [27] software.

nDNA Genotyping

Ten microsatellite loci were used in the analysis (Table 2) [28]. PCR amplification was performed in a total volume of 15 µl, with.5 mM $MgCl^2$, 0.5 mM dNTPs, 0.04 U/ml Taq DNA polymerase and 0.5 pmol/µl of each locus-specific primer with one of the primers in pair elongated for M13(-21) universal sequence (Schuelke 2000), 0.25 lM of M13(-21) universal primer labelled with dyes 6-FAM, VIC, PET or NED (Applied Biosys- tems, Foster City, CA, USA), 0.375 unit of Taq DNA polymerase (Fermentas, Vilnius, Lithuania) and 25 ng of genomic DNA. Fragment analysis by capillary electrophoresis was performed commercially at STAB VIDA Lda. Microsatellite genotypes were visualized and manually controlled with GeneMapper version 4.1 software (Applied Biosystems).

GenAlEx version 6.5 [29] was used to determine the following parameters of genetic variability: number of alleles (n), number of effective alleles (ne), observed heterozygosity (Ho), expected heterozygosity (He) and Unbiased Expected heterozygosity (uHe). POPGENE version 1.31 [29] was used to calculate deviations from Hardy–Weinberg equilibrium with likelihood ratio (G2 test) and for determination of the Shannon's Information Index (I) and fixation index (Fst) and inbreeding coefficient (Fis) and gene flow (Nm) between populations. The genetic distances were estimated on the same software using Nei's (1978) unbiased genetic distance coefficient, and the dendrogram was constructed based on the unweighted pair-group arithmetic average (UPGMA) method and visualized in Mega X [25]. STRUCTURE 2.3.4 [30] was used to classify the individuals into a set number of clusters (K). Using the admixture ancestry model and correlated allele frequency model different values for K, from one to ten, were tested running the analysis 10 times for each cluster (each run consisted of a burn-in period of 50 000 and 100 000 Markov chain Monte Carlo (MCMC) repetitions after the initial burn-in). The most appropriate cluster number was selected using the method reported by [31].

Table 2: Microsatellite [28] loci motif, accession numbers and primer used

Locus name	Motif	Acc. Number	Primer sequence
Boms5	$(CA)_{10}TA(CA)_2$ $(CA)_9$	EU489749	F: TGTAAAACGACGGCCAGTTCTCGCCCCAATTACCAC<br>R: GAATTTTGGCAACATCCAAGC
Boms8	$(CA)_7CG(CA)_5$	EU489752	F: TGTAAAACGACGGCCAGT TGACATACATGCCTTCATTCAC<br>R: CAGAAAAGCTTAAAACTAGCGG
Boms18	$(CA)_{13}$	AF467828	F: TGTAAAACGACGGCCAGTGCCATGAATGCAGACCAC<br>R: CCTATTCAAATGCACGCAAAAC
Boms21	$GTGG(GT)_{13}ATGT$	AF467827	F: TGTAAAACGACGGCCAGTTCGCCTCTTACCTCACAACC<br>R: ACCATCCTTAGTCAGCACAGTC
Boms25	$(GT)_{12}$	AF467826	F: TGTAAAACGACGGCCAGT TGGAATGCGCTATTTTGTTG<br>R: ACTCGTATATACGTACATGG
Boms30	$(GT)_{17}$	AF467823	F: TGTAAAACGACGGCCAGT CTGACTTCTTGCTTTACACG<br>R: CAGCTTATCTGCTTTAAGTGC
Boms32	$(CA)_{14}$	EU489765	F: TGTAAAACGACGGCCAGTTGTATGTATTTGTGCGTCG<br>R: GCTTAGACCATTTGCTCC
Boms34	$(CA)_3CTA(CA)_8$	EU489767	F: TGTAAAACGACGGCCAGTACGCCGCACACTTCTTAAAC<br>R: CACCCAACTTTTGTAGTTTCC
Boms58	$A_6CA_3GCA_6TA_5CA_5$	EU489782	F: TGTAAAACGACGGCCAGTAGTTGGACGCGCACATATC<br>R: AGCGCGTACGAGCTTTAGC
Boms59	$TGTA(TG)_{10}$	DQ078250	F: TGTAAAACGACGGCCAGTAGCGCTTACATAAATATAGCTAC<br>R: TCCCCGTAAAGCCATAAAGTC

Ca. Erwinia dacicola 16S sequences

For the selective amplification of a fragment sequence of the 16S recombinant deoxyribonucleic acid (rDNA) of *Ca*. Erwinia dacicola, a specific primer (EdF1) was paired with 1507R for PCR as previously described [11]. PCR reactions were conducted using 1 µl of the extracted DNA in a standard 25 µl reaction, with 0.5 pmol/µl of each primer, 1.5 mM $MgCl^2$, 0.5 mM dNTPs and 0.04 U/ml Taq DNA polymerase with the following cycle protocol: initial denaturation at 94 °C for 5 min, followed by 30 cycles of denaturation at 94 °C for 30 s, annealing for 30 s at 55 °C and extension at 72 °C for 30 s, followed by a final extension of 72 °C for 10 min. The PCR products, after visualized on agarose gel, were purified using the NZYGelpure kit (from NZYTech, Lda) and sequencing was done commercially (Macrogen Inc.). Electropherograms were inspected using Genestudio (www.genestudio.com). The evolutionary history was inferred by Maximum Likelihood and using the Hasegawa-Kishino-Yano model as selected model of evolution. Both analyses were performed in Mega X [25].

Results

From the sections of mtDNA sequenced, we retrieved a total of 3626 bp and 87 polymorphic sites (S2 Table) and Table 3 further summarizes nucleotide data at the four segments analysed, including Tajima's Neutrality Test that suggest that the DNA sequences studied are evolving under a non-random process, with many low frequency alleles causing low average divergence – characteristic of population expansion.

Table 3: Nucleotide frequencies and polymorphisms in *Bactrocera oleae* (S = number of segregating sites, ps = S/m, Θ = ps/a1, π = nucleotide diversity, and D is the Tajima test statistic);

	**Nucleotide Frequencies (%)**					**Tajima's Neutrality Test**				
gene	T	C	A	G	Total⁽²⁾	S	ps	Θ	π	D
**ND2**	36.5	18.4	35.1	9.9	1005.9	8	0.009	0.002	0.001	-1.537
**Cox**	33.6	20.6	28.9	16.9	605.1	14	0.023	0.005	0.002	-1.490
**CLC** (1)	33.7	21.4	32.3	12.6	989.4	39	0.039	0.008	0.003	-2.018
**ND4**	26.9	16.7	49.1	7.3	870.7	12	0.012	0.002	0.001	-1.154
**Total**	32.8	19.2	36.7	11.3	3471.1	88	0.024	0.005	0.002	-1.838

(1) fragment comprising part of COX1, tRNA-Leu (UUR) and COX2; (2) average number of total nucleotides

The Median-joining work representing relationships among mitochondrial haplotypes of olive fly is represented in Fig 1.

Fig 1. Median-joining network representing relationships among the haplotypes (S2 Table) from the analysed segments of mitochondrial DNA of the olive fruit fly (*Bactrocera oleae*). Circles represent haplotypes and the size of the circles is proportional to the frequency of the haplotype (black circles represent unobserved intermediate haplotypes and the length of the connections is proportional to the number of mutational steps that separate the haplotypes; these shared haplotypes are described on S3 Table). Colours correspond to the geographical cluster represented in the map. The grey coloured ovals correspond to maximum likelihood clades with a bootstrap value higher than 70% (in 1000 replicates; S4 Figure).

All ten microsatellite markers were polymorphic, revealing a total of 54 alleles with the parameters of genetic variability being presented on Table 4.

Table 4: Parameters of genetic variability: number of alleles (n), number of effective alleles (ne), observed heterozygosity (Ho), expected heterozygosity (He), Unbiased Expected heterozygosity (uHe), information index (I), Hardy–Weinberg equilibrium (HWE), fixation index (Fst) and inbreeding coefficient (FIS) and gene flow (Nm) among sampling sites.

	**N**	**ne**	**Ho**	**He**	**uHe**	**I**	**HWE (p)**	**Fst**	**Fis**	**Nm**
Boms5	5	1.762	0.398	0.418	0.443	0.814	0.07	0.035	0.048	6.971
Boms8	6	1.591	0.368	0.342	0.362	0.649	0.92	0.078	-0.076	2.972
Boms18	5	3.023	0.784	0.658	0.696	1.187	0.47	0.107	-0.191	2.084
Boms21	6	2.479	0.609	0.560	0.599	1.062	0.62	0.088	-0.087	2.577
Boms25	6	2.575	0.607	0.602	0.638	1.073	0.66	0.047	-0.010	5.034
Boms30	5	2.414	0.541	0.570	0.605	0.991	0.00	0.093	0.051	2.441
Boms32	8	4.089	0.852	0.748	0.795	1.537	0.40	0.049	-0.139	4.845
Boms34	4	2.250	0.524	0.543	0.576	0.932	0.46	0.067	0.035	3.494
Boms58	4	2.589	0.609	0.592	0.627	1.071	0.59	0.069	-0.028	3.396
Boms59	5	2.039	0.379	0.487	0.514	0.874	0.01	0.071	0.222	3.266

*p value ($P < 0.05$)

Genetic distances among samples from all the locations were measured according to Nei's unbiased genetic distance and the UPGMA dendrogram constructed suggests the grouping of data in two different clusters (Fig 2) albeit not related with a clear geographical pattern.

Fig 2: UPGMA dendrogram of the different sampled populations based on Nei's unbiased genetic distance; the color codes correspond to cluster colors on Fig 1. The associated table shows Nei's genetic identity values (upper diagonal) and pairwise population FST values (below diagonal).

Whereas the UPGMA dendogram by definition depicts a dichotomous structure, this should be interpreted only has similarities between the haplotypes as one cluster seems the most likely number of clusters (K) according to the STRUCTURE analysis (Fig 3).

Fig 3: Plot of mean ln P(X|K) over 10 independent runs for each K (K = 1–10) suggests a higher likelihood of one cluster only.

The 16S fragment sequenced of the symbiont *Ca*. Erwinia dacicola specimens showed only 20 parsimonious informative sites leading to a reconstructed phylogeny (Fig 4) where the two known haplotypes -htA and htB- are in a clade (two SNPs only) and represented by 69 and 14 sequences respectively (S3 File). Two new haplotypes, htC and htD with only 3 and 4 representatives each were found exclusively in Zarzis (htC) or Boufiche (htD) populations (note that these sequences were confirmed by two independent reactions of PCR and sanger sequencing). htC diverges from htA at 7 SNPs (and from htB at 9 SNPs), but also diverges from htD at 11 SNPs. The biggest pairwise differences are found between htD and htB (20 SNPs compared to the 18 SNPs with htA) (S5 File for sequence details).

Fig 4: Phylogenetic reconstruction of the *ca*. Erwinia dacicola fragment sequence (814 bp after quality trimming and alignment) of the 16S recombinant deoxyribonucleic acid (rDNA) gene. Three other *Erwinia* sequences (KC139453, *E. persicina*; KT766070 *E. aphidicola*; JX867759, *E. piriflorinigrans*) were used to root the phylogeny (not shown). The tree with the highest log likelihood (-1304.66) is shown together with the percentage of trees in which the associated taxa clustered together (next to the branches, 1000 replicates). The selected Hasegawa-Kishino-Yano model with a discrete Gamma distribution was used to model evolutionary rate differences among sites (+G, parameter = 0.05). The tree is drawn to scale, with branch lengths measured in the number of substitutions per site.

Discussion

The so far established structure of *B. oleae* in the Mediterranean Basin points to the existence of three clusters, with the olive fly populations in Iberia and the Levant differentiating from the ones of the Italic peninsula, albeit with intermixing [8,24]. The present work focused on the Iberian cluster and hypothesised that the Tunisian samples would fall into a differentiated cluster due to the proximity to the Italic peninsula cluster. The data obtained does not allow to confirm or dismiss the hypothesis, and a more detailed work with a higher number of samples and locations is needed. If something, the data on the fly suggests that population in this area is better seen as single cluster with no clear differentiation between populations. If for the mtDNA fragments the number of samples per population is not critical, it is well below the advisable number for microsatellite analyses (between 25 to 30 individuals seem to be needed for a reliable estimation of allele frequencies; [32]). Unfortunately, sampling relied on number of emerging adults from collected olive fruits and a high number of fruits render no insects, either by not having been infected or by death of the larvae due to storage and/or shipping conditions.

However, and when looking at the obligate symbiont *Ca.* Erwinia dacicola, two new 16S haplotypes were found exclusively in the populations from Tunisia. The olive fruit fly exhibits a strongest dependence of its endosymbiont *Ca.* E. dacicola, as it on its own does not possess the enzymatic systems required for feeding on unripped olive fruit. This actually means that the fly is a holobiont [33], better seen as a group of genetically different entities subjected to natural selection (at the level of holobiont

and hologenome rather than individuals or genomes [34]). This degree of dependence associated to a vertical mode of transmission strengthens the integrity of the holobiont and stresses the importance of looking at the genetic diversity of the obligate symbiont for our understanding of the host population dynamics.

The geographical data on the *Ca.* E. dacicola is still scarce. The present work duplicates the known number of 16S haplotypes. These new haplotypes seem to make a sister group with the two already described 16S haplotypes [35] (Fig 4), and so far they were only found south of western Mediterranean Basin. Two hypotheses could be put forward explaining these two sister groups: 1) a single event of symbiont acquisition followed by symbiont divergence through evolution, driven by geographical or functional determinants; 2) two events of symbiont acquisition, either unrelated or due to symbiont loss and de novo acquisition through a horizontal transmission event. The limited geographical data available does not allow to disentangle between the raised hypotheses.

Following the principle that "the simplest explanation is usually the best one", a single event of symbiont acquisition is the most likely hypothesis. In vertically transmitted symbionts there is a reduction in the purifying activity of natural selection ([36,37]) and the symbiont population is subjected to transmission bottlenecks, both with consequences on the symbiont genome. Theory predicts that strictly vertical transmitted symbionts are associated with high rates of amino acid substitution and the resulting reduced nucleotide base composition does not favour guanidine (G) and cytosine (C) (reviewed in [38]). Likewise, they show gene inactivation and loss, and ultimately a reduced genome ([38–40]). However, the data so far on *Ca.* Erwinia dacicola shows that both the genome size and GC content are similar to those of free-living bacteria than to those of other intracellular bacteria found in other insects [41,42]. These features are usually associated with facultative symbionts or with symbionts that only recently became obligate [43]. From what we know on this symbiosis, none of those special cases seem to apply. First, *Ca.* Erwinia dacicola seems to be an obligate symbiont, as it is believed that it allows the insect to overcome olive-plant secondary metabolites (and in particular the oleuropein) [6] and it is present in all life stages of wild olive fruit flies, being thus maintained through natural changes in diet and host metamorphosis [44]. Secondly, the olive fruit fly differentiation in the Mediterranean

seems to be connected with the post-glacial recolonization of wild olives in the area [45], suggesting that symbiont acquisition is also not recent as the fly seems to be long exploiting this olive fruit resource. In such a scenario, the 'Erwinia' transition into an obligate symbiont probably occurred far into the evolutionary history of the olive fruit fly, before the tree domestication begun. Note needs to be taken here, as experimental data have shown that the olive fruit fly can survive and reach adulthood on ripped fruit without the obligate symbiont [6]. This raises the alternative hypothesis of a recent shift of this 'Erwinia' species into an obligate, vertical transmitted symbiont, probably following the domestication and human-mediated spread of this culture. On the other hand, this bacterium was found to transition between intracellular and extracellular lifestyles during specific stages of the host's life cycle which together with the need to cope with a somewhat changing environment during the development of its polyphagous, holometabolous host, can be the reasons for the genomic similarity with free-living bacteria [11]. Also needing to be acknowledge is the possibility of DNA exchange with other transient gut bacteria (e.g., free living *Enterobacter* spp.) during the endosymbiont extracellular life stage, where they might coexist in the gut environment. Either way, a single transition to an obligate symbiont that became transmitted 100% vertically and uniparental (via the female) should lead to a structure genetically uniform in a clonal manner (clonality defined as in [46], the balance between vertical and horizontal gene inheritance amongst bacteria) purging genetic diversity from the population. Yet we now found a different *Ca.* E. dacicola 16S clade which on average diverges about 2% from the previously 16S known haplotypes. Only a study designed to disentangle the life history of these lineages will be able to distinguish between the different hypotheses, and in particularly between eventual recombination events, episodes of horizontal transmission of the symbiont or a 'de novo' acquisition event. An extracellular lifestyle of this symbiotic bacteria could indeed potentiate recombination events via coprophagy and/or the trophallaxis behaviour, as horizontal transfer of symbionts was observed in controlled conditions via cohabitation of symbiont-free lab populations and wild flies [47].

Nonetheless, we are dealing with a tripartite symbiosis with high specificities, and to understand olive fruit fly population diversity and dispersion there is a need to look at the olive tree population, its cultivars' diversity and specificities, and also to the symbiont that enables the fly to so efficiently and

uniquely exploit the fruit of the olive tree. All data suggest for high levels of intermixing of the fly populations, including a fast spreading of genes with fitness advantage (this ample gene flow is clear from the studies on the alleles associated with OPs resistance; [45,48,49]). The association between *B~~actrocera~~ oleae* and its obligate symbiont *Candidatus* Erwinia dacicola is key to success of the fly, enabling several generations per year as unripped fruit can be used successfully. Understanding this relation will aid in understanding pest dispersion and dynamics and in the search of alternative and sustainable pest management methods (symbiosis based; [50,51]).

Acknowledgements

I would like to deeply thank Imen Blibech for the samples from Tunisia, Raquel Garcia for the Montpellier samples, Juan Olivares and the Agropecuaria Vallefrío Nueva S.L. in La Roda (Albacete) for the Spanish samples and Fernando T. Rei for the Portuguese samples. Without their contribution, the present work would not have been possible.

Support

This work is supported by National Funds through FCT - Foundation for Science and Technology under the research project PTDC/ASP-PLA/30650/2017.

References

1. Lavee S, Zohary D. The potential of genetic diversity and the effect of geographically isolated resources in olive breeding. Isr J Plant Sci. 2011;59: 3–13. doi:10.1560/IJPS.59.1.3

2. Besnard G. The complex history of the olive tree : from Late Quaternary diversification of Mediterranean lineages to primary domestication in the northern Levant . Proc. R. Soc. B.

2013, doi:10.1098/rspb.2012.2833

3. Weiss E. Beginnings of fruit growing in the Old World " – two generations later. Isr J Plant Sci. 2015;62: 75–85. doi:10.1080/07929978.2015.1007718

4. Montiel A, Jones O. Alternative methods for controlling the olive fly. IOBC-WPRS Bull. 2001;25: 1–11.

5. Daane KM, Johnson MW. Olive fruit fly: managing an ancient pest in modern times. Annu Rev Entomol. 2010;55: 151–169. doi:10.1146/annurev.ento.54.110807.090553

6. Ben-Yosef M, Pasternak Z, Jurkevitch E, Yuval B. Symbiotic bacteria enable olive fly larvae to overcome host defences. R Soc Open Sci. 2015;2: 150170. doi:10.1098/rsos.150170

7. Capuzzo C, Firrao G, Mazzon L, Squartini A, Girolami V. "Candidatus Erwinia dacicola", a coevolved symbiotic bacterium of the olive fly Bactrocera oleae (Gmelin). Int J Syst Evol Microbiol. 2005;55: 1641–1647. doi:10.1099/ijs.0.63653-0

8. Van Asch B, Pereira-Castro I, Rei FT, Da Costa LT. Marked genetic differentiation between Western Iberian and Italic populations of the olive fly: Southern France as an intermediate area. PLoS One. 2015;10. doi:10.1371/journal.pone.0126702

9. Nardi F, Carapelli A, Dallai R, Roderick GK, Frati F. Population structure and colonization history of the olive fly, Bactrocera oleae (Diptera, Tephritidae). Mol Ecol. 2005;14: 2729–2738. doi:10.1111/j.1365-294X.2005.02610.x

10. van Asch B, Pereira-Castro I, Rei F, da Costa LT. Mitochondrial haplotypes reveal olive fly (Bactrocera oleae) population substructure in the Mediterranean. Genetica. 2012;140: 181–187. doi:10.1007/s10709-012-9669-2

11. Estes AM, Hearn DJ, Bronstein JL, Pierson EA. The olive fly endosymbiont, "Candidatus Erwinia dacicola," switches from an intracellular existence to an extracellular existence during host insect development. Appl Environ Microbiol. 2009;75: 7097–7106. doi:10.1128/AEM.00778-09

12. Petri L. Ricerche sopra i batteri intestinali della mosca olearia. Memorie della Regia Stazione di Patologia vegetale. Roma: Tipografia nazionale di G. Bertero; 1909. p. iv+129.

13. Sabater-Muñoz B, Toft C, Alvarez-Ponce D, Fares MA. Chance and necessity in the genome evolution of endosymbiotic bacteria of insects. ISME J. 2017;11: 1291–1304. doi:10.1038/ismej.2017.18

14. Savio C, Mazzon L, Martinez-Sañudo I, Simonato M, Squartini A, Girolami V, et al. Evidence of two lineages of the symbiont "Candidatus Erwinia dacicola" in Italian populations of Bactrocera oleae (Rossi) based on 16S rRNA gene sequences. Int J Syst Evol Microbiol. 2011;62: 179–187. doi:10.1099/ijs.0.030668-0

15. Ordano M, Engelhard I, Rempoulakis P, Nemny-Lavy E, Blum M, Yasin S, et al. Olive fruit fly (*Bactrocera oleae*) population dynamics in the Eastern Mediterranean: Influence of exogenous uncertainty on a monophagous frugivorous Insect. PLoS One. 2015;10: 1–18. doi:10.1371/journal.pone.0127798

16. Zalom FG, Burrack HJ, Bingham R, Price R, Ferguson L. Olive fruit fly introduction and establishment in California. Acta Horticulturae. International Society for Horticultural Science (ISHS), Leuven, Belgium; 2008. pp. 619–627. doi:10.17660/ActaHortic.2008.791.95

17. Malheiro R, Casal S, Baptista P, Pereira JA. A review of *Bactrocera oleae* (Rossi) impact in olive products: From the tree to the table. Trends Food Sci Technol. 2015;44: 226–242. doi:10.1016/j.tifs.2015.04.009

18. Cramer W, Guiot J, Fader M, Garrabou J, Gattuso J-PP, Iglesias A, et al. Climate change and interconnected risks to sustainable development in the Mediterranean. Nat Clim Chang. 2018;8: 972–980. doi:10.1038/s41558-018-0299-2

19. Battisti A, Larsson S. Climate change and insect pest distribution range. In: Björkman C, Niemelä P, editors. Climate Change and Insect Pests. CABI, Wallingford; 2015. pp. 1–15.

20. Furlong MJ, Zalucki MP. Climate change and biological control: the consequences of increasing temperatures on host–parasitoid interactions. Curr Opin Insect Sci. 2017;20: 39–44.

doi:10.1016/j.cois.2017.03.006

21. Thomson LJ, Macfadyen S, Hoffmann AA. Predicting the effects of climate change on natural enemies of agricultural pests. Biol Control. 2010;52: 296–306. doi:10.1016/j.biocontrol.2009.01.022

22. Castex V, Beniston M, Calanca P, Fleury D, Moreau J. Pest management under climate change : The importance of understanding tritrophic relations. Sci Total Environ. 2018;616–617: 397–407. doi:10.1016/j.scitotenv.2017.11.027

23. Hunt GJ. Insect DNA Extraction Protocol. In: Micheli MR, Bova R, editors. Fingerprinting Methods Based on Arbitrarily Primed PCR. Berlin, Heidelberg: Springer Berlin Heidelberg; 1997. pp. 21–24. doi:10.1007/978-3-642-60441-6_3

24. Nardi F, Carapelli A, Boore JL, Roderick GK, Dallai R, Frati F. Domestication of olive fly through a multi-regional host shift to cultivated olives: Comparative dating using complete mitochondrial genomes. Mol Phylogenet Evol. 2010;57: 678–686. doi:10.1016/j.ympev.2010.08.008

25. Kumar S, Stecher G, Li M, Knyaz C, Tamura K. MEGA X : Molecular Evolutionary Genetics Analysis across computing platforms. 2018;35: 1547–1549. doi:10.1093/molbev/msy096

26. Bandelt H.-J, Forster P, Rohl A. Median-Joining Networks for inferring intraspecific phylogenies. Molceular Biol Evol. 1999;16: 37–48.

27. Leigh JW, Bryant D. POPART : full-feature software for haplotype network construction. Methods inEcology andEvolution. 2015;6: 1110–1116. doi:10.1111/2041-210X.12410

28. Augustinos AA, Stratikopoulos EE, Drosopoulou E, Kakani EG, Mavragani-Tsipidou P, Zacharopoulou A, et al. Isolation and characterization of microsatellite markers from the olive fly, Bactrocera oleae, and their cross-species amplification in the Tephritidae family. BMC Genomics. 2008;9: 618. doi:10.1186/1471-2164-9-618

29. Peakall R, Smouse PE. GenAlEx 6 . 5 : genetic analysis in Excel . Population genetic software

for teaching and research — an update. Bioinformatics. 2012;28: 2537–2539. doi:10.1093/bioinformatics/bts460

30. Pritchard JK, Stephens M, Donnelly P. Inference of population structure using multilocus genotype data. Genetics. 2000;155: 945–959.

31. Evanno G, Regnaut S, Goudet J. Detecting the number of clusters of individuals using the software STRUCTURE : a simulation study. 2005. doi:10.1111/j.1365-294X.2005.02553.x

32. Hale ML, Burg TM, Steeves TE. Sampling for microsatellite-based population genetic studies : 25 to 30 individuals per population is enough to accurately estimate allele frequencies. 2012;7. doi:10.1371/journal.pone.0045170

33. Zilber-Rosenberg I, Rosenberg E. Role of microorganisms in the evolution of animals and plants: The hologenome theory of evolution. FEMS Microbiol Rev. 2008;32: 723–735. doi:10.1111/j.1574-6976.2008.00123.x

34. Gilbert SF, Sapp J, Tauber AI. A symbiotic view of life : We have never been individuals. Q Rev Biol. 2012;87: 325–341. doi:http://www.jstor.org/stable/10.1086/668166

35. Savio C, Mazzon L, Martinez-Sañudo I, Simonato M, Squartini A, Girolami V. Evidence of two lineages of the symbiont “Candidatus Erwinia dacicola” in Italian populations of Bactrocera oleae (Rossi) based on 16S rRNA gene sequences. Int J Syst Evol Microbiol. 2011;62: 179–187. doi:10.1099/ijs.0.030668-0

36. Funk DJ, Wernegreen JJ, Moran NA. Intraspecific variation in symbiont genomes : Bottlenecks and the Aphid-Buchnera association. Genetics. 2001;157: 477–489.

37. Mira A, Moran NA. Estimating population size and transmission bottlenecks in maternally transmitted endosymbiotic bacteria. Microb Ecol. 2002;44: 137–143. doi:10.1007/s00248-002-0012-9

38. Moran NA, Mccutcheon JP, Nakabachi A. Genomics and Evolution of Heritable Bacterial Symbionts. 2009. doi:10.1146/annurev.genet.41.110306.130119

39. Baumann P, Moran NA, Baumann L. Bacteriocyte-Associated Endosymbionts of Insects BT - The Prokaryotes: Volume 1: Symbiotic associations, Biotechnology, Applied Microbiology. In: Dworkin M, Falkow S, Rosenberg E, Schleifer K-H, Stackebrandt E, editors. New York, NY: Springer New York; 2006. pp. 403–438. doi:10.1007/0-387-30741-9_16

40. Moya A, Peretó J, Gil R, Latorre A. Learning how to live together: genomic insights into prokaryote-animal symbioses. Nat Rev Genet. 2008;9: 218–29. doi:10.1038/nrg2319

41. Estes AM, Hearn DJ, Nadendla S, Pierson EA, Dunning Hotopp JC. Draft genome sequence of Erwinia dacicola, a dominant endosymbiont of olive flies. Microbiol Resour Announc. 2018;7: 1–2. doi:10.1128/MRA.01067-18

42. Blow F, Gioti A, Starns D, Ben-Yosef M, Pasternak Z, Jurkevitch E, et al. Draft genome sequence of the *Bactrocera oleae* symbiont "*Candidatus* Erwinia dacicola." Genome Announc. 2016;4: e00896-16. doi:10.1128/genomeA.00896-16

43. Bright M, Bulgheresi S. A complex journey : transmission of microbial symbionts. Nat Rev Microbiol. 2010;8: 218–230. doi:10.1038/nrmicro2262.A

44. Estes AM, Hearn DJ, Burrack HJ, Rempoulakis P, Pierson EA. Prevalence of *Candidatus* Erwinia dacicola in wild and laboratory olive fruit fly populations and across developmental stages. Environ Entomol. 2012;41: 265–274. doi:10.1603/EN11245

45. Nardi F, Carapelli A, Vontas JG, Dallai R, Roderick GK, Frati F. Geographical distribution and evolutionary history of organophosphate-resistant Ace alleles in the olive fly (*Bactrocera oleae*). Insect Biochem Mol Biol. 2006;36: 593–602. doi:10.1016/j.ibmb.2006.05.002

46. Shapiro BJ. How clonal are bacteria over time? Curr Opin Microbiol. 2016;31: 116–123. doi:10.1016/j.mib.2016.03.013

47. Bigiotti G, Pastorelli R, Guidi R, Belcari A, Sacchetti P. Horizontal transfer and finalization of a reliable detection method for the olive fruit fly endosymbiont , *Candidatus* Erwinia dacicola. BMC Biotechnol. 2019;19: 1–12. doi:10.1186/s12896-019-0583-x

48. Nobre T, Gomes L, Rei FT. A re-rvaluation of olive fruit fly organophosphate-resistant Ace alleles in Iberia, and field-testing population effects after in-practice dimethoate use. Insects. 2019;10: 1–10.

49. Pereira-Castro I, Van Asch B, Trinidade Rei F, Texeira Da Costa L. *Bactrocera oleae* (Diptera: Tephritidae) organophosphate resistance alleles in Iberia: Recent expansion and variable frequencies. Eur J Entomol. 2015;112: 20–26. doi:10.14411/eje.2015.019

50. Nobre T. Symbiosis in sustainable agriculture: Can olive fruit fly bacterial microbiome be useful in pest management? Microorganisms. 2019;7: 238. doi:10.3390/microorganisms7080238

51. Apostolaki A, Livadaras I, Saridaki A, Chrysargyris A, Savakis C, Bourtzis K. Transinfection of the olive fruit fly *Bactrocera oleae* with *Wolbachia*: Towards a symbiont-based population control strategy. J Appl Entomol. 2011;135: 546–553. doi:10.1111/j.1439-0418.2011.01614.x

Supporting information

S1 Table. Primers used to amplify Bactrocera oleae mitochondrial DNA segments analysed in this study.

S2 Table. Sequence variability having the *Bactrocera oleae* mitochondrion, complete genome AY210702 as reference

S3 Table. Mitochondrial haplotypes shared by different specimens

S4 Fig. *Bactrocera oleae* maximum likelihood reconstruction

S5 File 16S variable sites and haplotype inference

Fig 1

Click here to access/download;Figure;Figure 1new.tif

Figure 1.tif

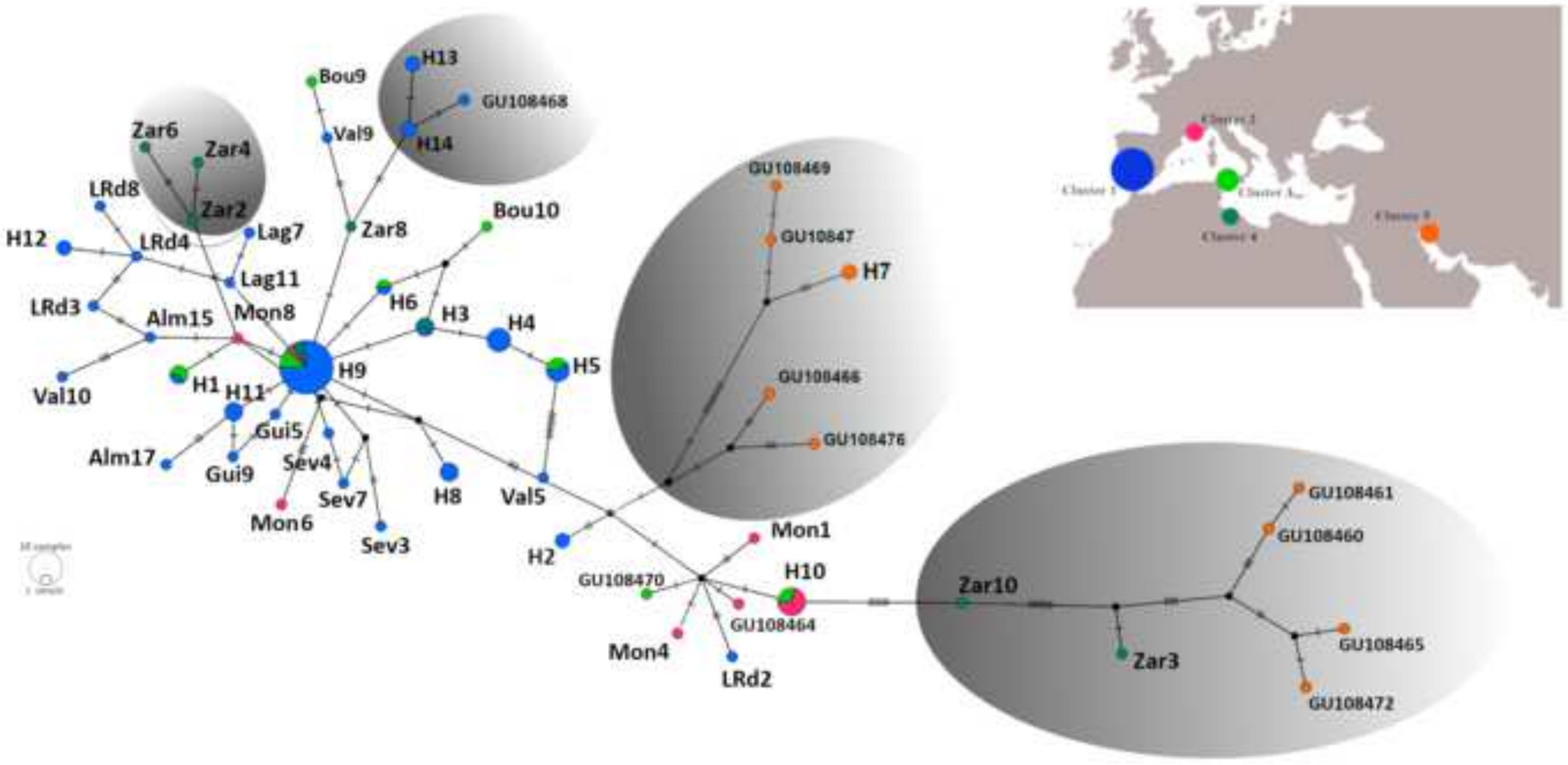

Fig 2

Click here to access/download;Figure;Figure 2new.tif

Figure 2.tif

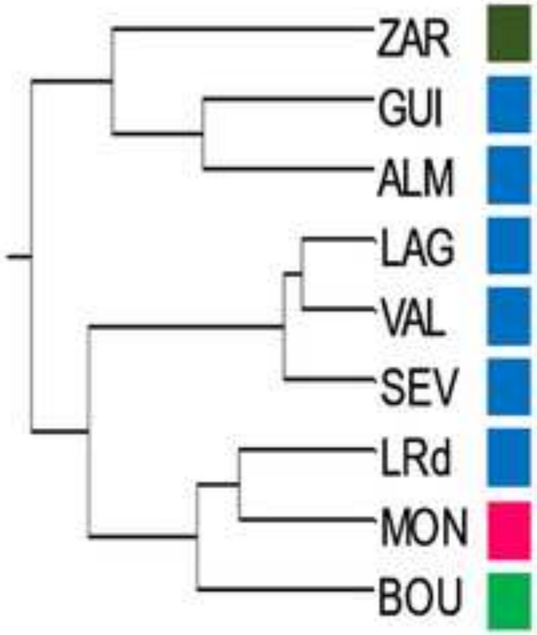

ZAR	GUI	ALM	LAG	VAL	SEV	LRD	MON	BOU
*	0.882	0.898	0.874	0.887	0.875	0.869	0.875	0.839
0.044	*	0.922	0.924	0.906	0.852	0.878	0.881	0.900
0.036	0.031	*	0.928	0.895	0.857	0.878	0.865	0.893
0.045	0.032	0.029	*	0.937	0.935	0.911	0.924	0.895
0.039	0.039	0.039	0.024	*	0.930	0.914	0.911	0.901
0.047	0.060	0.053	0.024	0.029	*	0.876	0.891	0.831
0.047	0.048	0.044	0.030	0.030	0.043	*	0.909	0.922
0.046	0.049	0.051	0.026	0.032	0.038	0.032	*	0.920
0.058	0.040	0.043	0.039	0.037	0.060	0.030	0.030	*

Fig 3

Click here to access/download;Figure;Figure 3.tif

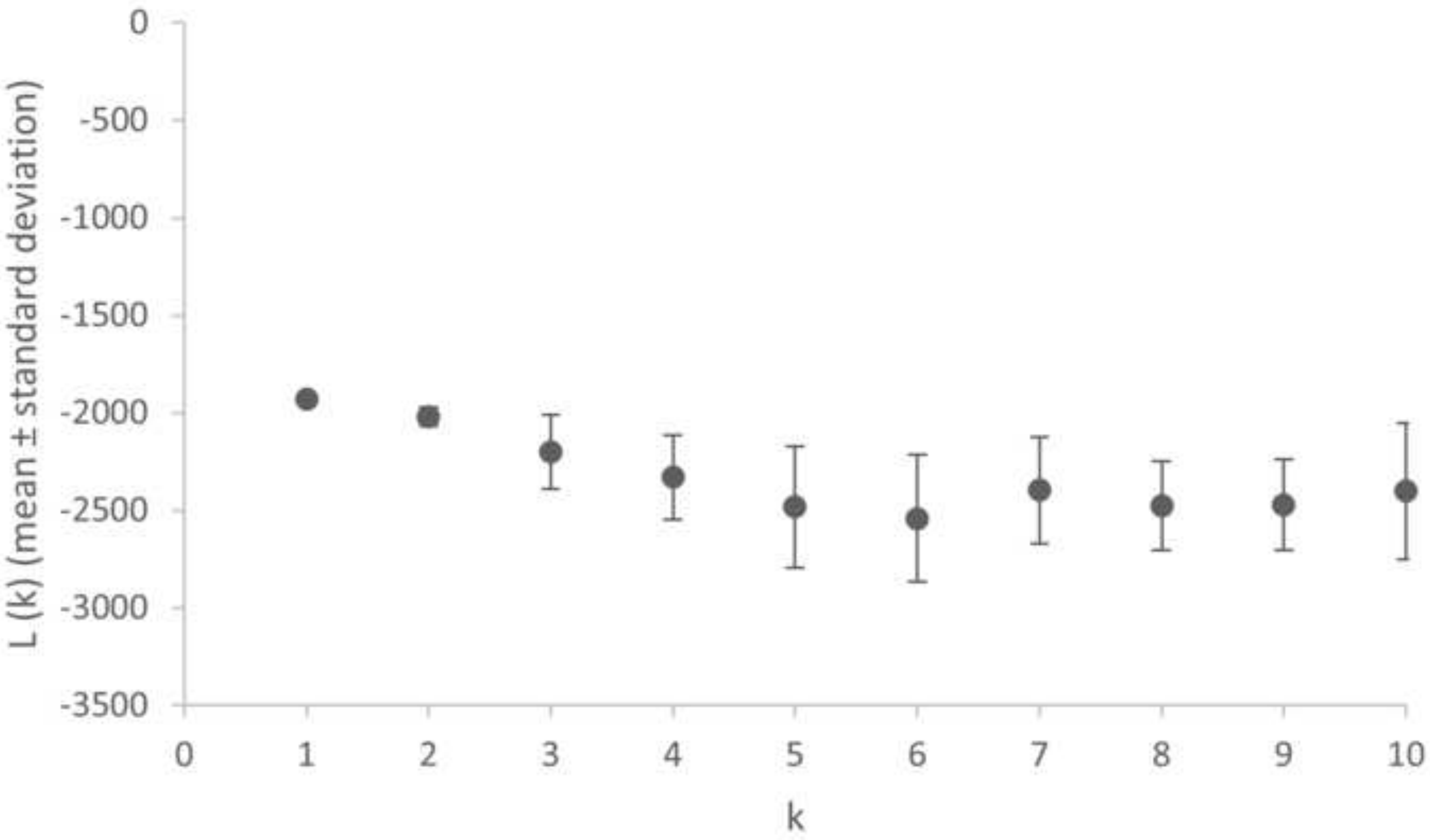

 Click here to access/download;Figure;Figure 4.tif

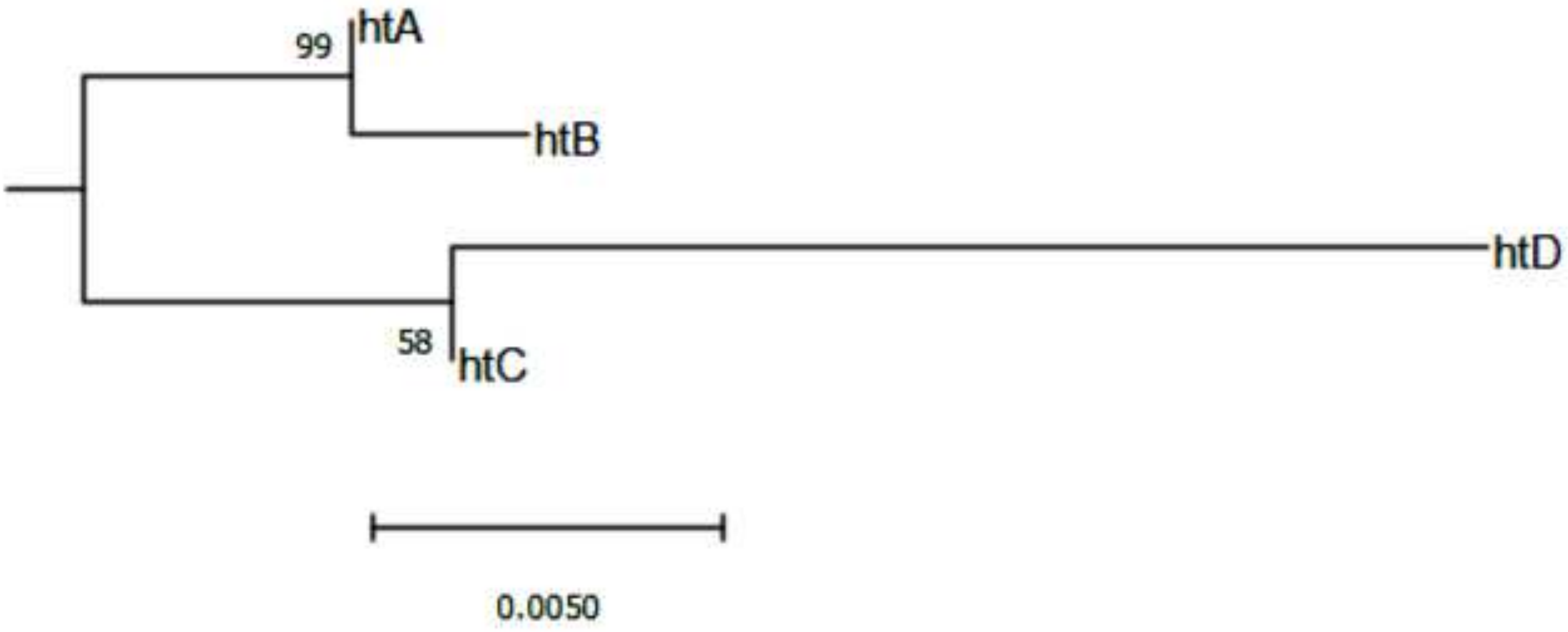

Primers used to amplify Bactrocera oleae mitochondrial DNA segments analysed in this study.

Click here to access/download
Supporting Information
S1 Table.xls

Sequence variability having the Bactrocera oleae mitochondrion, complete genome AY210702 as reference.

Click here to access/download
Supporting Information
S2 Table.xls

Mitochondrial haplotypes shared by different specimens.

Click here to access/download
Supporting Information
S3 Table.xls

Bactrocera oleae maximum likelihood reconstruction.

Click here to access/download
Supporting Information
S4 Fig.docx

16S variable sites and haplotype inference

Click here to access/download
Supporting Information
S5 File.xls

Olive fruit fly and its obligate symbiont *Candidatus* Erwinia dacicola: two new symbiont haplotypes in the Mediterranean basin

Tânia Nobre*

MED - Mediterranean Institute for Agriculture, Environment and Development, Instituto de Investigação e Formação Avançada, Universidade de Évora, Pólo da Mitra, Ap. 94, 7006-554 Évora, Portugal.

* Corresponding author

Email: tnobre@uevora.pt

Abstract

The olive fruit fly, specialized to become monophagous during several life stages, remains the most important olive tree pest with high direct production losses, but also affecting the quality, composition, and inherent properties of the olives. Thought to have originated in Africa is nowadays present wherever olive groves are grown. The olive fruit fly evolved to harbor a vertically transmitted and obligate bacterial symbiont ~~–~~ -*Candidatus* Erwinia dacicola- leading thus to a tight evolutionary history between olive tree, fruit fly and obligate, vertical transmitted symbiotic bacterium. Considering this linkage, the genetic diversity (at a 16S fragment) of this obligate symbiont was added in the understanding of the distribution pattern of the holobiont at nine locations throughout four countries in the Mediterranean Basin. This was complemented with mitochondrial (four mtDNA fragments) and nuclear (ten microsatellites) data of the host. We focused on the previously established Iberian cluster for the *B. oleae* structure and hypothesised that the Tunisian samples would fall into a differentiated cluster. From the host point of view, we were unable to confirm this hypothesis. ~~but l~~Looking at the symbiont, however, two new 16S haplotypes were found~~,~~ exclusively in the populations from Tunisia. This finding is discussed in the frame of host-symbiont specificity and transmission mode. To understand olive fruit fly population diversity and dispersion, the dynamics of the symbiont also needs to be taken into consideration, as it ~~that~~ enables the fly to, so efficiently and uniquely, exploit ~~this~~ the olive fruit resource ~~also need to be taken into consideration~~.

Keywords: pest management; olive pest; *Bactrocera oleae*; holobiont; symbiont transmission

Introduction

The olive tree was likely the first domesticated fruit tree, and although domestication probably began in the Eastern Mediterranean, selection for cultivars took place at several different independent

locations [1–3]. Given time and appropriate conditions, the olive fruit fly evolved to explore this resource. This fruit fly, *Bactrocera oleae* (Rossi, 1790), has specialized to become monophagous and it remains the most important olive tree pest. Production losses are estimated on an average of more than 15% yearly [4], and this fly has been responsible for losses of up to 80% of oil value and 100% of some table cultivars [5]. To cope with the olive-plant abundant secondary metabolites, particularly the defensive compound oleuropein (a bitter and otherwise toxic phenolic glycoside) [6], the olive fruit fly evolved to harbor a vertically transmitted and obligate bacterial symbiont—*Candidatus* Erwinia dacicola [7] leading thus to a tight evolutionary history between olive tree, fruit fly and obligate, vertical transmitted symbiotic bacterium.

The olive fruit fly is thought to have originated in Africa and then spread to the Mediterranean basin and South Central Asia [8], and it is nowadays present wherever olive groves are grown. Nardi and co-workers [9] presented the first comprehensive study on olive fly populations and found evidence of a population subdivision into three regional groups, Pakistan, Africa and Mediterranean plus America. Further, there is evidence of a separation between Iberian (Western Mediterranean) and Italic (Central Mediterranean) olive fly populations [10], with no clearly defined boundary [8]. Van Asch and co-workers [8] observed intermixing to extend at variable levels throughout their whole studied area, from Northwestern Italy to Portugal.

As an obligate symbiont, mechanisms that ensure vertical transmission have evolved and *Ca.* Erwinia dacicola is transmitted to the following generation at the oviposition [7,11,12] guaranteeing symbiont acquisition by the offspring but also host-symbiont specificity. When transmission is strictly vertical, host switching would not be expected, and co-evolution of host and symbiont lineages should be observed. For both symbiont and host, failure in vertical transmission has high costs, and is usually equivalent to death of the symbiont and significant reduction in host fitness or high mortality. Selection at these endosymbiotic bacteria likely results from selection imposed by the host and that from selection emerging in a symbiotic context but independent from the host [13]. Furthermore, the cyclic events of transmission -where a given number of symbionts are passed to the next generation- are likely to create strong bottlenecks affecting symbiont population dynamics. Not much is known on the diversity and

structure of *ca.* Erwinia dacicola except that there is evidence of two lineages of this endosymbiont in Italian populations, based on 16S rRNA gene sequences [14].

Understanding how the pest population spreads is essential for its management. The close association of this monophagous fly species with its host, and its possibility of laying eggs throughout most of the year, allows for a fairly stable population and the rate of population change is mainly shaped by density-dependent population feedback [15]. Its spreading is intricately connected to the spreading of the plant host, as this relation seems the result of long-term co-evolution. In most areas where olives are grown commercially, the olive fruit fly can be found and have become a major pest. In North America was first detected in 1998 in California [16], and it has spread throughout the region, including in Central and South America. Nowadays it seems that the olive fruit fly can also be found well outside the Mediterranean basin in Central and South Africa, and not only in the Middle East but also in China and India; Australia seems to be the only area in the world, where olives are cultivated and well established for commercial purposes, which is still free of olive fruit fly [17].

Having as rationality this intricate tripartite relation between olive tree, olive fruit fly and *Ca*. Erwinia dacicola symbiont, this work aimed to add this obligate symbiont genetic diversity in the understanding of the distribution pattern of the holobiont at nine locations throughout four countries in the Mediterranean Basin. We focused on the Iberian cluster and on the hypothesis that the Tunisian samples would fall into a differentiated cluster due to the proximity to the Italic peninsula cluster. To date, and to the best of our knowledge, the genetic diversity present within the olive fly symbiont has only investigated for the Italian populations [14]. Therefore, the present study looked at a) haplotypes from four mitochondrial DNA fragments of the host fly, b) patterns of ten microsatellites of the host fly and c) 16S rRNA gene sequence diversity of *Ca.* E. dacicola. Albeit with a limited geographical and populational coverage, it was intended to look for population differentiation, gaining insights into gene flow and spreading. Overall rising temperatures, warmer winter minimum temperatures and changes in precipitation patterns that are likely linked to water shortages [18] are affecting the spreading of insects and their aggressiveness as pests in largely unknown ways [19]. These changes will lead to species

specific dynamics [20–22], which likely will ~~with~~ impact on the interaction of the olive and its obligate olive fruit fly.

Methodology

Formatted: English (United Kingdom)

Data collection

Olive fruit flies were sampled at 9 locations (3 in Portugal, 3 in Spain, 1 in France and 2 in Tunisia; Table 1), either directly, with the permission of the owners, or made available by colleagues and/or landowners (see acknowledgements). Sampling did not involve endangered or protected species. Olives were collected and stored in plastic boxes, with emerging pupae and adults being gathered up to 12 specimens per location. Individuals were stored at -20 °C in 70 % ethanol until DNA extraction. Individuals were allowed to dry on filter paper prior to DNA extraction. DNA from whole body tissue was extracted following extraction protocols [23] using CTAB extraction buffer after being ground up with a plastic pestle. Proteins were removed with 24 : 1 isoamylalcohol : chloroform, and DNA precipitated with isopropanol. DNA extracts were eluted in 50 μ~~L~~l of sterile water. All extraction products were stored at -20 °C and later used directly in the PCRs.

Table 1: Sampling locations and collection year (see acknowledgements for collectors' information)

Country	Location	Code	Coordinates		Year
Portugal	Guimarães	GUI	41.46N	8.31W	2016
Portugal	Valverde	VAL	38.86N	7.27W	2019
Portugal	Lagos	LAG	37.13N	8.68W	2016
Spain	LaRoda	LRD	39.09N	2.19W	2019
Spain	Seville	SEV	37.47N	5.99W	2019
Spain	Almeria	ALM	36.89N	2.44W	2016
France	Montpellier	MON	43.61N	3.87E	2017
Tunisia	Boufícha	BOU	36.30N	10.45E	2017
Tunisia	Zarzis	ZAR	33.50N	11.12E	2017

Olive fruit fly mtDNA sequences

Four highly variable sections of mtDNA were amplified and sequenced. These were amongst the five sections selected by van Asch and co-workers [10], based on the number of polymorphisms previously

described in a Mediterranean region dataset [24]. These include both tRNA-Leu genes and segments of the ND2, ND4, COX1 and~~,~~ COX2 and ~~16srRNA~~ genes, ca. 24% of the complete mitochondrial genome of *B. oleae* [10] (see S1 Table for primer information). PCR reactions were conducted using 1 µl of the extracted DNA in a standard 25 µl reaction, with 0.5 pmol/µl of each primer, 1.5 mM $MgCl^2$, 0.5 mM dNTPs and 0.04 U/ml Taq DNA polymerase. The touch-down cycle protocol involved two-phases; 1) initial denaturation at 94 °C for 5 min, followed by 10 cycles of denaturation at 94 °C for 30 s, annealing at variable temperatures for 30 s (set at 60 °C and decreasing by 0.5 °C per cycle) and extension at 72 °C for 1 min; 2) 25 cycles of 94°C for 30 s, 50 °C for 30 s, and 72°C for 1 min, followed by a final extension of 72 °C for 7 min. The PCR products, after visualized on agarose gel, were purified using the NZYGelpure kit (from NZYTech, Lda) and sequencing was done commercially (Macrogen Inc.).

Electropherograms were inspected using Genestudio V.2.2.0.0 (www.genestudio.com) and sequences were cropped to the minimal region of overlap for all individuals using the same software. The 21 *Bactrocera oleae* full mitochondrial genomes made available by Nardi and co-workers [24] were aligned with our sequences and the same regions of overlap were extracted. All alignments were made using the Muscle tool implemented in MEGA X [25]. The amplified mitochondrial DNA segments and the database obtained ones were concatenated per individual. Median-joining networks [26] were calculated using the PopART [27] software.

nDNA Genotyping

Ten microsatellite loci were used in the analysis (Table 2) [28]. PCR amplification was performed in a total volume of 15 µl, with.5 mM $MgCl^2$, 0.5 mM dNTPs, 0.04 U/ml Taq DNA polymerase and 0.5 pmol/µl of each locus-specific primer with one of the primers in pair elongated for M13(-21) universal sequence (Schuelke 2000), 0.25 lM of M13(-21) universal primer labelled with dyes 6-FAM, VIC, PET or NED (Applied Biosys- tems, Foster City, CA, USA), 0.375 unit of Taq DNA polymerase (Fermentas, Vilnius, Lithuania) and 25 ng of genomic DNA. Fragment analysis by capillary electrophoresis was performed commercially at STAB VIDA Lda. Microsatellite genotypes were

visualized and manually controlled with GeneMapper version 4.1 software (Applied Biosystems). GenAlEx version 6.5 [29] was used to determine the following parameters of genetic variability: number of alleles (n), number of effective alleles (ne), observed heterozygosity (Ho), expected heterozygosity (He) and Unbiased Expected heterozygosity (uHe). POPGENE version 1.31 [29] was used to calculate deviations from Hardy–Weinberg equilibrium with likelihood ratio (G2 test) and for determination of the Shannon's Information Index (I) and fixation index (Fst) and inbreeding coefficient (Fis) and gene flow (Nm) between populations. The genetic distances were estimated on the same software using Nei's (1978) unbiased genetic distance coefficient, and the dendrogram was constructed based on the unweighted pair-group arithmetic average (UPGMA) method and visualized in Mega X [25]. STRUCTURE 2.3.4 [30] was used to classify the individuals into a set number of clusters (K). Using the admixture ancestry model and correlated allele frequency model different values for K, from one to ten, were tested running the analysis 10 times for each cluster (each run consisted of a burn-in period of 50 000 and 100 000 Markov chain Monte Carlo (MCMC) repetitions after the initial burn-in). The most appropriate cluster number was selected using the method reported by [31].

Table 2: Microsatellite [28] loci motif, accession numbers and primer used

Locus name	Motif	Acc. Number	Primer sequence
Boms5	$(CA)_{10}TA(CA)_2$ $(CA)_9$	EU489749	F: TGTAAAACGACGGCCAGTTCTCGCCCCAATTACCAC<br>R: GAATTTTGGCAACATCCAAGC
Boms8	$(CA)_7CG(CA)_5$	EU489752	F: TGTAAAACGACGGCCAGT TGACATACATGCCTTCATTCAC<br>R: CAGAAAAGCTTAAAACTAGCGG
Boms18	$(CA)_{13}$	AF467828	F: TGTAAAACGACGGCCAGTGCCATGAATGCAGACCAC<br>R: CCTATTCAAATGCACGCAAAAC
Boms21	$GTGG(GT)_{13}ATGT$	AF467827	F: TGTAAAACGACGGCCAGTTCGCCTCTTACCTCACAACC<br>R: ACCATCCTTAGTCAGCACAGTC
Boms25	$(GT)_{12}$	AF467826	F: TGTAAAACGACGGCCAGT TGGAATGCGCTATTTTGTTG<br>R: ACTCGTATATACGTACATGG
Boms30	$(GT)_{17}$	AF467823	F: TGTAAAACGACGGCCAGT CTGACTTCTTGCTTTACACG<br>R: CAGCTTATCTGCTTTAAGTGC
Boms32	$(CA)_{14}$	EU489765	F: TGTAAAACGACGGCCAGTTGTATGTATTTGTGCGTCG<br>R: GCTTAGACCATTTGCTCC
Boms34	$(CA)_3CTA(CA)_8$	EU489767	F: TGTAAAACGACGGCCAGTACGCCGCACACTTCTTAAAC<br>R: CACCCAACTTTTGTAGTTTCC
Boms58	$A_6CA_3GCA_6TA_5CA_5$	EU489782	F: TGTAAAACGACGGCCAGTAGTTGGACGCGCACATATC<br>R: AGCGCGTACGAGCTTTAGC
Boms59	$TGTA(TG)_{10}$	DQ078250	F: TGTAAAACGACGGCCAGTAGCGCTTACATAAATATAGCTAC<br>R: TCCCCGTAAAGCCATAAAGTC

Ca. Erwinia dacicola 16S sequences

For the selective amplification of a fragment sequence of the 16S recombinant deoxyribonucleic acid (rDNA) of *Ca.* Erwinia dacicola, a specific primer (EdF1) was paired with 1507R for PCR as previously described [11]. PCR reactions were conducted using 1 µl of the extracted DNA in a standard 25 µl reaction, with 0.5 pmol/µl of each primer, 1.5 mM $MgCl^2$, 0.5 mM dNTPs and 0.04 U/ml Taq DNA polymerase with the following cycle protocol: initial denaturation at 94 °C for 5 min, followed by 30 cycles of denaturation at 94 °C for 30 s, annealing for 30 s at 55 °C and extension at 72 °C for 30 s, followed by a final extension of 72 °C for 10 min. The PCR products, after visualized on agarose gel, were purified using the NZYGelpure kit (from NZYTech, Lda) and sequencing was done commercially (Macrogen Inc.). Electropherograms were inspected using Genestudio (www.genestudio.com). The evolutionary history was inferred by Maximum Likelihood and using the Hasegawa-Kishino-Yano model as selected model of evolution. Both analyses were performed in Mega X [25].

Results

From the sections of mtDNA sequenced, we retrieved a total of 3626 bp and 87 polymorphic sites (S2~~1~~ Table) and Table 3 further summarizes nucleotide data at the four segments analysed, including Tajima's Neutrality Test that suggest that the DNA sequences studied are evolving under a non-random process, with many low frequency alleles causing low average divergence – characteristic of population expansion.

Table 3: Nucleotide frequencies and polymorphisms in *Bactrocera oleae* (S = number of segregating sites, ps = S/m, Θ = ps/a1, π = nucleotide diversity, and D is the Tajima test statistic);

	**Nucleotide Frequencies (%)**					**Tajima's Neutrality Test**				
gene	T	C	A	G	Total(2)	S	ps	Θ	π	D
**ND2**	36.5	18.4	35.1	9.9	1005.9	8	0.009	0.002	0.001	-1.537
**Cox**	33.6	20.6	28.9	16.9	605.1	14	0.023	0.005	0.002	-1.490
**CLC** (1)	33.7	21.4	32.3	12.6	989.4	39	0.039	0.008	0.003	-2.018
**ND4**	26.9	16.7	49.1	7.3	870.7	12	0.012	0.002	0.001	-1.154
**Total**	32.8	19.2	36.7	11.3	3471.1	88	0.024	0.005	0.002	-1.838

(1) fragment comprising part of COX1, tRNA-Leu (UUR) and COX2; (2) average number of total nucleotides

The Median-joining work representing relationships among mitochondrial haplotypes of olive fly is represented in Fig 1.

Fig 1. Median-joining network representing relationships among the haplotypes (S2 Table) from the analysed segments of mitochondrial DNA of the olive fruit fly (*Bactrocera oleae*). Circles represent haplotypes and the size of the circles is proportional to the frequency of the haplotype (black circles represent unobserved intermediate haplotypes and the length of the connections is proportional to the number of mutational steps that separate the haplotypes~~)~~; these shared haplotypes are described on S3 Table). Colours correspond to the geographical cluster represented in the map. The grey coloured ovals correspond to maximum likelihood clades with a bootstrap value higher than 70% (in 1000 replicates; ~~S2~~ S4 Figure).

Formatted: Not Highlight

All ten microsatellite markers were polymorphic, revealing a total of 54 alleles with the parameters of genetic variability being presented on Table 4.

Table 4: Parameters of genetic variability: number of alleles (n), number of effective alleles (ne), observed heterozygosity (Ho), expected heterozygosity (He), Unbiased Expected heterozygosity (uHe), information index (I), Hardy–Weinberg equilibrium (HWE), fixation index (Fst) and inbreeding coefficient (FIS) and gene flow (Nm) among sampling sites.

	**N**	**ne**	**Ho**	**He**	**uHe**	**I**	**HWE (p)**	**Fst**	**Fis**	**Nm**
Boms5	5	1.762	0.398	0.418	0.443	0.814	0.07	0.035	0.048	6.971
Boms8	6	1.591	0.368	0.342	0.362	0.649	0.92	0.078	-0.076	2.972
Boms18	5	3.023	0.784	0.658	0.696	1.187	0.47	0.107	-0.191	2.084
Boms21	6	2.479	0.609	0.560	0.599	1.062	0.62	0.088	-0.087	2.577
Boms25	6	2.575	0.607	0.602	0.638	1.073	0.66	0.047	-0.010	5.034
Boms30	5	2.414	0.541	0.570	0.605	0.991	0.00	0.093	0.051	2.441
Boms32	8	4.089	0.852	0.748	0.795	1.537	0.40	0.049	-0.139	4.845
Boms34	4	2.250	0.524	0.543	0.576	0.932	0.46	0.067	0.035	3.494
Boms58	4	2.589	0.609	0.592	0.627	1.071	0.59	0.069	-0.028	3.396
Boms59	5	2.039	0.379	0.487	0.514	0.874	0.01	0.071	0.222	3.266

*p value ($P < 0.05$)

Genetic distances among samples from all the locations were measured according to Nei's unbiased genetic distance and the UPGMA dendrogram constructed suggests the grouping of data in two different clusters (Fig 2) albeit not related with a clear geographical pattern.

Fig 2: UPGMA dendrogram of the different sampled populations based on Nei's unbiased genetic distance; the color codes correspond to cluster colors on Fig 1. The associated table shows Nei's genetic identity values (upper diagonal) and pairwise population FST values (below diagonal).

~~However~~Whereas the UPGMA dendogram by definition depicts a dichotomous structure, this should be interpreted only has similarities between the haplotypes as~~,~~ one cluster seems the most likely number of clusters (K) according to the STRUCTURE analysis (Fig 3).

Fig 3: Plot of mean ln P(X|K) over 10 independent runs for each K (K = 1–10) suggests a higher likelihood of one cluster only.

The 16S fragment sequenced of the symbiont *Ca*. Erwinia dacicola specimens showed only 20 parsimonious informative sites leading to a reconstructed phylogeny (Fig 4) where the two known haplotypes -htA and htB- are in a clade (two SNPs only) and represented by 69 and 14 sequences respectively (S3 File). Two new haplotypes, htC and htD with only 3 and 4 representatives each were found exclusively in Zarzis (htC) or Boufiche (htD) populations (note that these sequences were confirmed by two independent reactions of PCR and sanger sequencing). htC diverges from htA at 7 SNPs (and from htB at 9 SNPs), but also diverges from htD at 11 SNPs. The biggest pairwise differences are found between htD and htB (20 SNPs compared to the 18 SNPs with htA) (~~S3~~ S5 File for sequence details).

Formatted: Font: 11 pt

Fig 4: Phylogenetic reconstruction of the *ca*. Erwinia dacicola fragment sequence (814 bp after quality trimming and alignment) of the 16S recombinant deoxyribonucleic acid (rDNA) gene. Three other *Erwinia* sequences (KC139453, *E. persicina*; KT766070 *E. aphidicola*; JX867759, *E. piriflorinigrans*) were used to root the phylogeny (not shown). The tree with the highest log likelihood (-1304.66) is shown together with the percentage of trees in which the associated taxa clustered together (next to the branches, 1000 replicates). The selected Hasegawa-Kishino-Yano model with a discrete Gamma distribution was used to model evolutionary rate differences among sites (+G, parameter = 0.05). The tree is drawn to scale, with branch lengths measured in the number of substitutions per site.

Discussion

The so far established structure of *B. oleae* in the Mediterranean Basin points to the existence of three clusters, with the olive fly populations in Iberia and the Levant differentiating from the ones of the Italic peninsula, albeit with intermixing [8,24]. The present work focused on the Iberian cluster and hypothesised that the Tunisian samples would fall into a differentiated cluster due to the proximity to the Italic peninsula cluster. The data obtained does not allow to confirm or dismiss the hypothesis, and a more detailed work with a higher number of samples and locations is needed. If something, the data on the fly suggests that population in this area is better seen as single cluster with no clear differentiation between populations. If for the mtDNA fragments the number of samples per population is not critical, it is well below the advisable number for microsatellite analyses (between 25 to 30 individuals seem to be needed for a reliable estimation of allele frequencies; [32]). Unfortunately, sampling relied on number of emerging adults from collected olive fruits and a high number of fruits render no insects, either by not having been infected or by death of the larvae due to storage and/or shipping conditions.

However, and when looking at the obligate symbiont *Ca.* Erwinia dacicola, two new 16S haplotypes were found exclusively in the populations from Tunisia. The olive fruit fly exhibits a strongest dependence of its endosymbiont *Ca.* E. dacicola, as it on its own does not possess the enzymatic systems required for feeding on unripped olive fruit. This actually means that the fly is a holobiont [33], better seen as a group of genetically different entities subjected to natural selection (at the level of holobiont

and hologenome rather than individuals or genomes [34]). This degree of dependence associated to a vertical mode of transmission strengthens the integrity of the holobiont and stresses the importance of looking at the genetic diversity of the obligate symbiont for our understanding of the host population dynamics.

The geographical data on the *Ca.* E. dacicola is still scarce. The present work duplicates the known number of 16S haplotypes. These new haplotypes seem to make a sister group with the two already described 16S haplotypes [35] (Fig 4), and so far they were only found south of western Mediterranean Basin. Two hypotheses could be put forward explaining these two sister groups: 1) a single event of symbiont acquisition followed by symbiont divergence through evolution, driven by geographical or functional determinants; 2) two events of symbiont acquisition, either unrelated or due to symbiont loss and de novo acquisition through a horizontal transmission event. The limited geographical data available does not allow to disentangle between the raised hypotheses.

Following the principle that “the simplest explanation is usually the best one”~~parsimony~~, a single event of symbiont acquisition is the most likely hypothesis. In vertically transmitted symbionts there is a reduction in the purifying activity of natural selection (~~e.g.~~ [36,37]) and the symbiont population is subjected to transmission bottlenecks, both with consequences on the symbiont genome. Theory predicts that strictly vertical transmitted symbionts are associated with high rates of amino acid substitution and the resulting reduced nucleotide base composition does not favour guanidine (G) and cytosine (C) (reviewed in [38]). Likewise, they show gene inactivation and loss, and ultimately a reduced genome (~~e.g.~~ [38–40]). However, the data so far on *Ca.* Erwinia dacicola shows that both the genome size and GC content are similar to those of free-living bacteria than to those of other intracellular bacteria found in other insects [41,42]. These features are usually associated with facultative symbionts or with symbionts that only recently became obligate [43]. From what we know on this symbiosis, none of those special cases seem to apply~~ to this olive fruit fly symbiont~~. First, *Ca.* Erwinia dacicola seems to be an obligate symbiont, as it is believed that it allows the insect to overcome olive-plant secondary metabolites (and in particular the oleuropein) [6] and it is present in all life stages of wild olive fruit flies, being thus maintained through natural changes in diet and host metamorphosis

[44]. Secondly, the olive fruit fly differentiation in the Mediterranean seems to be connected with the post-glacial recolonization of wild olives in the area [45], suggesting that symbiont acquisition is also not recent as the fly seems to be long exploiting this olive fruit resource. In such a scenario, the ‘Erwinia’ transition into an obligate symbiont probably occurred far into the evolutionary history of the olive fruit fly, before the tree domestication begun. Note needs to be taken here, as experimental data have shown that the olive fruit fly can survive and reach adulthood on ripped fruit without the obligate symbiont [6]. This raises the alternative hypothesis of a recent shift of this ‘Erwinia’ species into an obligate, vertical transmitted symbiont, probably following the domestication and human-mediated spread of this culture. On the other hand, this ~~bacteria~~ bacterium was found to transition between intracellular and extracellular lifestyles during specific stages of the host’s life cycle which together with the need to cope with a somewhat changing environment during the development of its polyphagous, holometabolous host, can be the reasons for the genomic similarity with free-living bacteria [11]. Also needing to be acknowledge is the possibility of DNA exchange with other transient gut bacteria (e.g., free living *Enterobacter* spp.) during the endosymbiont extracellular life stage, where they might coexist in the gut environment. Either way, a single transition to an obligate symbiont that became transmitted 100% vertically and uniparental (via the female) should lead to a structure genetically uniform in a clonal manner (clonality defined as in [46], the balance between vertical and horizontal gene inheritance amongst bacteria) purging genetic diversity from the population. Yet we now found a different *Ca*. E. dacicola 16S clade which on average diverges about 2% from the previously 16S known haplotypes. Only a study designed to disentangle the life history of the~~i~~se lineages will be able to distinguish between the different hypothese~~i~~s, and in particularly between eventual recombination events, episodes of horizontal transmission of the symbiont or a ‘de novo’ acquisition event. An extracellular lifestyle of this symbiotic bacteria could indeed potentiate recombination events via coprophagy and/or the trophallaxis behaviour, as horizontal transfer of symbionts was observed in controlled conditions via cohabitation of symbiont-free lab populations and wild flies [47].

Nonetheless, we are dealing with a tripartite symbiosis with high specificities, and to understand olive fruit fly population diversity and dispersion there is a need to look at the olive tree population, its

Formatted: Font: Italic

cultivars' diversity and specificities, and also to the symbiont that enables the fly to so efficiently and uniquely exploit ~~this resource~~the fruit of the olive tree. All data suggest~~s~~ for high levels of intermixing of the fly populations, including a fast spreading of genes with fitness advantage (this ample gene flow is clear from the studies on the alleles associated with OPs resistance; ~~e.g.~~ [45,48,49]). The association between *Bactrocera oleae* and its obligate symbiont *Candidatus* Erwinia dacicola is key to success of the fly, enabling several generations per year as unripped fruit can be used successfully. Understanding this relation will aid in understanding pest dispersion and dynamics and in the search of alternative and sustainable pest management methods (~~e.g.~~ symbiosis based; [50,51]).

Acknowledgements

I would like to deeply thank Imen Blibech for the samples from Tunisia, Raquel Garcia for the Montpellier samples, Juan Olivares and the Agropecuaria Vallefrío Nueva S.L. in La Roda (Albacete) for the Spanish samples and Fernando T. Rei for the Portuguese samples. Without their contribution, the present work would not have been possible.

Support

This work is supported by National Funds through FCT - Foundation for Science and Technology under the research project PTDC/ASP-PLA/30650/2017.

References

1. Lavee S, Zohary D. The potential of genetic diversity and the effect of geographically isolated resources in olive breeding. Isr J Plant Sci. 2011;59: 3–13. doi:10.1560/IJPS.59.1.3
2. Besnard G. The complex history of the olive tree : from Late Quaternary diversification of

Mediterranean lineages to primary domestication in the northern Levant . Proc. R. Soc. B. 2013, doi:10.1098/rspb.2012.2833

3. Weiss E. Beginnings of fruit growing in the Old World ” – two generations later. Isr J Plant Sci. 2015;62: 75–85. doi:10.1080/07929978.2015.1007718

4. Montiel A, Jones O. Alternative methods for controlling the olive fly. IOBC-WPRS Bull. 2001;25: 1–11.

5. Daane KM, Johnson MW. Olive fruit fly: managing an ancient pest in modern times. Annu Rev Entomol. 2010;55: 151–169. doi:10.1146/annurev.ento.54.110807.090553

6. Ben-Yosef M, Pasternak Z, Jurkevitch E, Yuval B. Symbiotic bacteria enable olive fly larvae to overcome host defences. R Soc Open Sci. 2015;2: 150170. doi:10.1098/rsos.150170

7. Capuzzo C, Firrao G, Mazzon L, Squartini A, Girolami V. “Candidatus Erwinia dacicola”, a coevolved symbiotic bacterium of the olive fly Bactrocera oleae (Gmelin). Int J Syst Evol Microbiol. 2005;55: 1641–1647. doi:10.1099/ijs.0.63653-0

8. Van Asch B, Pereira-Castro I, Rei FT, Da Costa LT. Marked genetic differentiation between Western Iberian and Italic populations of the olive fly: Southern France as an intermediate area. PLoS One. 2015;10. doi:10.1371/journal.pone.0126702

9. Nardi F, Carapelli A, Dallai R, Roderick GK, Frati F. Population structure and colonization history of the olive fly, Bactrocera oleae (Diptera, Tephritidae). Mol Ecol. 2005;14: 2729–2738. doi:10.1111/j.1365-294X.2005.02610.x

10. van Asch B, Pereira-Castro I, Rei F, da Costa LT. Mitochondrial haplotypes reveal olive fly (Bactrocera oleae) population substructure in the Mediterranean. Genetica. 2012;140: 181–187. doi:10.1007/s10709-012-9669-2

11. Estes AM, Hearn DJ, Bronstein JL, Pierson EA. The olive fly endosymbiont, “Candidatus Erwinia dacicola,” switches from an intracellular existence to an extracellular existence during host insect development. Appl Environ Microbiol. 2009;75: 7097–7106.

doi:10.1128/AEM.00778-09

12. Petri L. Ricerche sopra i batteri intestinali della mosca olearia. Memorie della Regia Stazione di Patologia vegetale. Roma: Tipografia nazionale di G. Bertero; 1909. p. iv+129.

13. Sabater-Muñoz B, Toft C, Alvarez-Ponce D, Fares MA. Chance and necessity in the genome evolution of endosymbiotic bacteria of insects. ISME J. 2017;11: 1291–1304. doi:10.1038/ismej.2017.18

14. Savio C, Mazzon L, Martinez-Sañudo I, Simonato M, Squartini A, Girolami V, et al. Evidence of two lineages of the symbiont “Candidatus Erwinia dacicola” in Italian populations of Bactrocera oleae (Rossi) based on 16S rRNA gene sequences. Int J Syst Evol Microbiol. 2011;62: 179–187. doi:10.1099/ijs.0.030668-0

15. Ordano M, Engelhard I, Rempoulakis P, Nemny-Lavy E, Blum M, Yasin S, et al. Olive fruit fly (*Bactrocera oleae*) population dynamics in the Eastern Mediterranean: Influence of exogenous uncertainty on a monophagous frugivorous Insect. PLoS One. 2015;10: 1–18. doi:10.1371/journal.pone.0127798

16. Zalom FG, Burrack HJ, Bingham R, Price R, Ferguson L. Olive fruit fly introduction and establishment in California. Acta Horticulturae. International Society for Horticultural Science (ISHS), Leuven, Belgium; 2008. pp. 619–627. doi:10.17660/ActaHortic.2008.791.95

17. Malheiro R, Casal S, Baptista P, Pereira JA. A review of *Bactrocera oleae* (Rossi) impact in olive products: From the tree to the table. Trends Food Sci Technol. 2015;44: 226–242. doi:10.1016/j.tifs.2015.04.009

18. Cramer W, Guiot J, Fader M, Garrabou J, Gattuso J-PP, Iglesias A, et al. Climate change and interconnected risks to sustainable development in the Mediterranean. Nat Clim Chang. 2018;8: 972–980. doi:10.1038/s41558-018-0299-2

19. Battisti A, Larsson S. Climate change and insect pest distribution range. In: Björkman C, Niemelä P, editors. Climate Change and Insect Pests. CABI, Wallingford; 2015. pp. 1–15.

Formatted: Font: Italic

Formatted: Font: Italic

20. Furlong MJ, Zalucki MP. Climate change and biological control: the consequences of increasing temperatures on host–parasitoid interactions. Curr Opin Insect Sci. 2017;20: 39–44. doi:10.1016/j.cois.2017.03.006

21. Thomson LJ, Macfadyen S, Hoffmann AA. Predicting the effects of climate change on natural enemies of agricultural pests. Biol Control. 2010;52: 296–306. doi:10.1016/j.biocontrol.2009.01.022

22. Castex V, Beniston M, Calanca P, Fleury D, Moreau J. Pest management under climate change : The importance of understanding tritrophic relations. Sci Total Environ. 2018;616–617: 397–407. doi:10.1016/j.scitotenv.2017.11.027

23. Hunt GJ. Insect DNA Extraction Protocol. In: Micheli MR, Bova R, editors. Fingerprinting Methods Based on Arbitrarily Primed PCR. Berlin, Heidelberg: Springer Berlin Heidelberg; 1997. pp. 21–24. doi:10.1007/978-3-642-60441-6_3

24. Nardi F, Carapelli A, Boore JL, Roderick GK, Dallai R, Frati F. Domestication of olive fly through a multi-regional host shift to cultivated olives: Comparative dating using complete mitochondrial genomes. Mol Phylogenet Evol. 2010;57: 678–686. doi:10.1016/j.ympev.2010.08.008

25. Kumar S, Stecher G, Li M, Knyaz C, Tamura K. MEGA X : Molecular Evolutionary Genetics Analysis across ~~Computing~~ computing ~~P~~platforms. 2018;35: 1547–1549. doi:10.1093/molbev/msy096

26. Bandelt H.-J, Forster P, Rohl A. Median-Joining Networks for ~~Inferring~~ inferring ~~Intraspecific~~ intraspecific p~~P~~hylogenies. Molceular Biol Evol. 1999;16: 37–48.

27. Leigh JW, Bryant D. POPART : full-feature software for haplotype network construction. Methods inEcology andEvolution. 2015;6: 1110–1116. doi:10.1111/2041-210X.12410

28. Augustinos AA, Stratikopoulos EE, Drosopoulou E, Kakani EG, Mavragani-Tsipidou P, Zacharopoulou A, et al. Isolation and characterization of microsatellite markers from the olive fly, Bactrocera oleae, and their cross-species amplification in the Tephritidae family. BMC

Genomics. 2008;9: 618. doi:10.1186/1471-2164-9-618

29. Peakall R, Smouse PE. GenAlEx 6 . 5 : genetic analysis in Excel . Population genetic software for teaching and research — an update. Bioinformatics. 2012;28: 2537–2539. doi:10.1093/bioinformatics/bts460

30. Pritchard JK, Stephens M, Donnelly P. Inference of population structure using multilocus genotype data. Genetics. 2000;155: 945–959.

31. Evanno G, Regnaut S, Goudet J. Detecting the number of clusters of individuals using the software STRUCTURE : a simulation study. 2005. doi:10.1111/j.1365-294X.2005.02553.x

32. Hale ML, Burg TM, Steeves TE. Sampling for ~~Microsatellite~~microsatellite-~~Based~~ based p~~P~~opulation g~~G~~enetic s~~S~~tudies : 25 to 30 i~~I~~ndividuals per p~~P~~opulation i~~I~~s e~~E~~nough to a~~A~~ccurately e~~E~~stimate a~~A~~llele f~~F~~requencies. 2012;7. doi:10.1371/journal.pone.0045170

33. Zilber-Rosenberg I, Rosenberg E. Role of microorganisms in the evolution of animals and plants: The hologenome theory of evolution. FEMS Microbiol Rev. 2008;32: 723–735. doi:10.1111/j.1574-6976.2008.00123.x

34. Gilbert SF, Sapp J, Tauber AI. A symbiotic view of life : We have never been individuals. Q Rev Biol. 2012;87: 325–341. doi:http://www.jstor.org/stable/10.1086/668166

35. Savio C, Mazzon L, Martinez-Sañudo I, Simonato M, Squartini A, Girolami V. Evidence of two lineages of the symbiont “Candidatus Erwinia dacicola” in Italian populations of Bactrocera oleae (Rossi) based on 16S rRNA gene sequences. Int J Syst Evol Microbiol. 2011;62: 179–187. doi:10.1099/ijs.0.030668-0

36. Funk DJ, Wernegreen JJ, Moran NA. Intraspecific ~~Variation~~ variation in ~~Symbiont~~ symbiont ~~Genomes~~ genomes : Bottlenecks and the Aphid-Buchnera a~~A~~ssociation. Genetics. 2001;157: 477–489.

37. Mira A, Moran NA. Estimating ~~P~~population s~~S~~ize and ~~T~~transmission ~~Bottlenecks~~ bottlenecks in m~~M~~aternally t~~T~~ransmitted ~~E~~endosymbiotic ~~B~~bacteria. Microb Ecol. 2002;44: 137–143.

doi:10.1007/s00248-002-0012-9

38. Moran NA, Mccutcheon JP, Nakabachi A. Genomics and Evolution of Heritable Bacterial Symbionts. 2009. doi:10.1146/annurev.genet.41.110306.130119

39. Baumann P, Moran NA, Baumann L. Bacteriocyte-Associated Endosymbionts of Insects BT - The Prokaryotes: Volume 1: Symbiotic associations, Biotechnology, Applied Microbiology. In: Dworkin M, Falkow S, Rosenberg E, Schleifer K-H, Stackebrandt E, editors. New York, NY: Springer New York; 2006. pp. 403–438. doi:10.1007/0-387-30741-9_16

40. Moya A, Peretó J, Gil R, Latorre A. Learning how to live together: genomic insights into prokaryote-animal symbioses. Nat Rev Genet. 2008;9: 218–29. doi:10.1038/nrg2319

41. Estes AM, Hearn DJ, Nadendla S, Pierson EA, Dunning Hotopp JC. Draft genome sequence of Erwinia dacicola, a dominant endosymbiont of olive flies. Microbiol Resour Announc. 2018;7: 1–2. doi:10.1128/MRA.01067-18

42. Blow F, Gioti A, Starns D, Ben-Yosef M, Pasternak Z, Jurkevitch E, et al. Draft genome sequence of the *Bactrocera oleae* symbiont "*Candidatus* Erwinia dacicola." Genome Announc. 2016;4: e00896-16. doi:10.1128/genomeA.00896-16

43. Bright M, Bulgheresi S. A complex journey : transmission of microbial symbionts. Nat Rev Microbiol. 2010;8: 218–230. doi:10.1038/nrmicro2262.A

44. Estes AM, Hearn DJ, Burrack HJ, Rempoulakis P, Pierson EA. Prevalence of ~~candidatus~~ *Candidatus* Erwinia dacicola in wild and laboratory olive fruit fly populations and across developmental stages. Environ Entomol. 2012;41: 265–274. doi:10.1603/EN11245

45. Nardi F, Carapelli A, Vontas JG, Dallai R, Roderick GK, Frati F. Geographical distribution and evolutionary history of organophosphate-resistant Ace alleles in the olive fly (*Bactrocera oleae*). Insect Biochem Mol Biol. 2006;36: 593–602. doi:10.1016/j.ibmb.2006.05.002

46. Shapiro BJ. How clonal are bacteria over time? Curr Opin Microbiol. 2016;31: 116–123. doi:10.1016/j.mib.2016.03.013

Formatted: Font: Italic

Formatted: Font: Italic

Formatted: Font: Italic

Formatted: Font: Italic

47. Bigiotti G, Pastorelli R, Guidi R, Belcari A, Sacchetti P. Horizontal transfer and finalization of a reliable detection method for the olive fruit fly endosymbiont , *Candidatus* Erwinia dacicola. BMC Biotechnol. 2019;19: 1–12. doi:10.1186/s12896-019-0583-x

48. Nobre T, Gomes L, Rei FT. A Rere-rEvaluation of oOlive fFruit Ffly oOrganophosphate-rResistant Ace aAlleles in Iberia, and fField-tTesting pPopulation E eff-ects after in-pPractice dDimethoate uUse. Insects. 2019;10: 1–10.

49. Pereira-Castro I, Van Asch B, Trinidade Rei F, Texeira Da Costa L. *Bactrocera oleae* (Diptera: Tephritidae) organophosphate resistance alleles in Iberia: Recent expansion and variable frequencies. Eur J Entomol. 2015;112: 20–26. doi:10.14411/eje.2015.019

50. Nobre T. Symbiosis in sustainable agriculture: Can olive fruit fly bacterial microbiome be useful in pest management? Microorganisms. 2019;7: 238. doi:10.3390/microorganisms7080238

51. Apostolaki A, Livadaras I, Saridaki A, Chrysargyris A, Savakis C, Bourtzis K. Transinfection of the olive fruit fly *Bactrocera oleae* with *Wolbachia*: Towards a symbiont-based population control strategy. J Appl Entomol. 2011;135: 546–553. doi:10.1111/j.1439-0418.2011.01614.x

Supporting information

S1 Table. Primers used to amplify Bactrocera oleae mitochondrial DNA segments analysed in this study.

S2 Table. Sequence variability having the *Bactrocera oleae* mitochondrion, complete genome AY210702 as reference

S3 Table. Mitochondrial haplotypes shared by different specimens

S42 Fig. *Bactrocera oleae* maximum likelihood reconstruction

S3 S5 File 16S variable sites and haplotype inference

Formatted: Font: Italic

Formatted: Font: Italic

Formatted: Font: Italic

Formatted: Font: Italic

Formatted: Font: Not Bold

Dear editor

Thank you and the reviewers for the comments on our manuscript. We have revised the text, according to the recommendations of the reviewers, discussing the issues raised also in the manuscript. Below, we give a detailed response to all points raised by the reviewers. We hope that our revised manuscript can now be published in PloS One.

Sincerely,

Tânia Nobre

Reviewer #1: The author followed a holistic approach combining data from both the host and the endosymbiont. Interestingly two new 16S haplotypes regarding the Candidatus Erwnia dacicola were detected for the Tunisian olive fly populations, which is the major finding of the manuscript, adding valuable data in the existing findings regarding the endosymbiont genetic variability. There is also an interesting overall commentary regarding the olive fly population diversity and coevolution with the symbiont.

Generally, the manuscript is well written and structured employing a wide list of genomic tools and approaches. There is a main disadvantage regarding the microsatellites part with a restricted number of flies used for the analysis in order to have a reliable estimation. Author has explained the reason behind this and therefore assumptions based on microsatellites throughout the text are minimum.

There is a short list of minor issues that I would like to be addressed through a revised version of the manuscript:

A supplemental list with the primers/probes used would be beneficial for the reader.

This list was added as supplemental file.

Please do not use “e.g.” for citing references within the text.

Removed.

Line 87-88: “These changes will lead to species specific dynamics with impact on the interaction of the olive and its obligate olive fruit fly”. This is a strong statement and it should be followed by a list of relevant references.

Three relevant references were added to the text, that show that climate change will impact differently different insect species.

Line 249: “following the parsimony” please explain the meaning here and if necessary add references to support it.

The sentence was changed for better accuracy. What was meant as the principle of parsimony was the principle that the simplest explanation is usually the best one.

Line 270: correct “this bacteria was found”

Corrected

Line 287: “uniquely exploit this resource” where do you refer by “resource”?

By resource I meant the fruit of the olive tree. Sentence was changed accordingly

Line 287: please correct “all data suggests” to “all data suggest”.

Corrected

Reviewer #2: PONE-D-21-11736: Olive fruit fly and its obligate symbiont Candidatus Erwinia dacicola: two new symbiont haplotypes in the Mediterranean basin

This paper identifies two new haplotypes of the olive fly symbiont, Ca. Erwinia dacicola, found to be uniquely associated with flies from Tunisia, and significantly different from previously described haplotypes associated with samples from Europe. These findings contrast analyses based on genomic and mitochondrial markers suggesting that the populations sampled from Africa and Europe cluster with no clear association to their geographic location. The main conclusion is that since the symbiont is vertically transmitted and essential to its host the new bacterial linages may contribute to understanding the dispersion patterns and population diversity of the fly.

The paper is well written but please note that there are many remaining typos, syntax and parsing issues which need to be attended. From the perspective of my field of expertise I have only a few comments, mostly concerning topics addressed in the results and discussion. Please make sure that the sequencing data underlying the findings will be made publically available (I couldn't find a note on this in the manuscript).

The sequencing data on the olive fruit fly are directly available from the S1 Table as it shows all the nucleotide variability in relation to *Bactrocera oleae* mitochondrion, complete genome AY210702 as reference. The four Ca. Erwinia dacicola 16S haplotypes are also derived from the data made available on S3 File and were also submitted to GenBank under the codes htA: MW888710, htD: MW888711, htB: MW888712 and htC: MW888713.

Introduction:

Page 4, Lines 83-84 and 222-224 (discussion): please state the hypotheses of this study in the introduction as well.

Thank you. This was done and can now be read on Lines 79-80.

Results:

1. Figure 1 depicts the median-joining network of haplotypes derived from mitochondrial DNA analysis. In addition to the populations sampled in this study (9 locations), other flies seem to have been included in the analysis (e.g. H, GU, orange coded), which do not seem to be mentioned in the text. Please include a description of these samples. Mont = MON?

Thanks for the comment, which highlights that the information might not have been clear. The other sequences included are referred in the methodology L-119 to L-121 and these are represented by the GenBank codes and H# represents a haplotype shared by different samples. This information is indeed only presented in (S1 Table) and the haplotypes needed to be derived. Therefore, I added a S2 Table, facilitating the interpretation. A sentence is added to the legend now, for clarification. Mont = MON (now corrected).

2. In Figure 2 the color codes for Montpelier and Bouficha seem to have been mixed (they do not match those in figure 1). Should there be a representation of the orange-coded cluster as well (as in figure 1)?

You are absolutely correct as to the color codes of MON and BOU. Thank you. They are now corrected. Unfortunately, no representative of orange-coded cluster from figure 1 was

available in the sampled specimens (they all corresponded to sequences coming from previous study) and therefore they could not be included in the microsatellite analyses.

3. The STRUCTURE analysis and figure 3: this is a bit confusing. Should the microsatellite-based dendogram depict two clusters (as currently presented) or only one cluster? Please include a short clarification for readers which are unfamiliar with the technicalities of this specific test.

The UPGMA dendogram by definition depicts a dichotomous structure whereas STRUCTURE infers the most likely number of clusters for a given data set using a Bayesian approach. A sentence was added to aid the reader, as suggested.

Discussion:
The discussion addresses several issues but is currently presented as one continuous mass of text. I suggest to divide the text into clear sections complying with the addressed subjects.

Thank you for the suggestion. I did struggle with finding clear sections complying with the results addressed because my original effort was exactly the opposite: I have attempted to give a more holistic perspective to the discussion by combining data from both the host and the endosymbiont. I do believe that this is one of the strong points of the manuscript, also recognized by reviewer one. Therefore, I decided to stay with my original approach.

Lines 245 – 248: according to file S3, flies sampled from Tunisia (and other locations as well) can be associated with either one of the 4 identified symbiont haplotypes. Two points which might be considered to be included in the discussion: (1) Can the above be congruent with these two hypotheses?, and (2) can certain fly genotypes (e.g. mitochondrial haplotypes) be more prone to be associated with a certain bacterial haplotype?

No doubt are the points raised interesting and relevant. Unfortunately, the scarce geographical data available does not allow to dismiss none of the two hypotheses raised in the main text. Also, the interesting possibility of high host-symbiont haplotype specificity is not possible to address with the current data. However, as soon as more data is available, I believe these suggestions can and should be discussed. A sentence was added to the discussion to allocate this comment and the data limitation.

Line 268: I would separate the evolution of Erwinia into an obligate symbiont from the domestication of the olive. The transition into an obligate symbiont probably occurred far into the evolutionary history of the fly, and is not associated with recent human activity.

This was presented in the discussion as the main hypothesis, in lines 264-267. However, it seems that it was not clear enough and therefore a sentence was added for clarification. Also further on the sentence was re-written to accommodate the comment and becoming clearer.

Lines 269 – 273: another possible explanation contributing to retention of genome size of the Erwinia symbiont: it is a gut bacterium, and as such it remains exposed to transient bacteria which are ingested with the diet (probably mainly at the adult stage), and thus, to horizontal

gene transfer. The composition of the gut microbiome of this fly suggest that bacteria other than the Erwinia symbiont (e.g. free living Enterobacter spp.) are intermittently associated with the fly and may contribute to DNA exchange.

This hypothesis is now stated in a clearer way (Line 276-279).
